# Multimer Formation Explains Allelic Suppression of PRDM9 Recombination Hotspots

**DOI:** 10.1371/journal.pgen.1005512

**Published:** 2015-09-14

**Authors:** Christopher L. Baker, Pavlina Petkova, Michael Walker, Petr Flachs, Ondrej Mihola, Zdenek Trachtulec, Petko M. Petkov, Kenneth Paigen

**Affiliations:** 1 Center for Genome Dynamics, The Jackson Laboratory, Bar Harbor, Maine, United States of America; 2 Laboratory of Germ Cell Development, Division BIOCEV, Institute of Molecular Genetics of the Academy of Sciences of the Czech Republic, v. v. i., Prague, Czech Republic; Stowers Institute for Medical Research, UNITED STATES

## Abstract

Genetic recombination during meiosis functions to increase genetic diversity, promotes elimination of deleterious alleles, and helps assure proper segregation of chromatids. Mammalian recombination events are concentrated at specialized sites, termed hotspots, whose locations are determined by PRDM9, a zinc finger DNA-binding histone methyltransferase. *Prdm9* is highly polymorphic with most alleles activating their own set of hotspots. In populations exhibiting high frequencies of heterozygosity, questions remain about the influences different alleles have in heterozygous individuals where the two variant forms of PRDM9 typically do not activate equivalent populations of hotspots. We now find that, in addition to activating its own hotspots, the presence of one *Prdm9* allele can modify the activity of hotspots activated by the other allele. PRDM9 function is also dosage sensitive; *Prdm9* ^*+/-*^ heterozygous null mice have reduced numbers and less active hotspots and increased numbers of aberrant germ cells. In mice carrying two *Prdm9* alleles, there is allelic competition; the stronger *Prdm9* allele can partially or entirely suppress chromatin modification and recombination at hotspots of the weaker allele. In cell cultures, PRDM9 protein variants form functional heteromeric complexes which can bind hotspots sequences. When a heteromeric complex binds at a hotspot of one PRDM9 variant, the other PRDM9 variant, which would otherwise not bind, can still methylate hotspot nucleosomes. We propose that in heterozygous individuals the underlying molecular mechanism of allelic suppression results from formation of PRDM9 heteromers, where the DNA binding activity of one protein variant dominantly directs recombination initiation towards its own hotspots, effectively titrating down recombination by the other protein variant. In natural populations with many heterozygous individuals, allelic competition will influence the recombination landscape.

## Author Summary

During formation of sperm and eggs chromosomes exchange DNA in a process known as recombination, creating new combinations responsible for much of the enormous diversity in populations. In some mammals, including humans, the locations of recombination are chosen by a DNA-binding protein named PRDM9. Importantly, there are tens to hundreds of different variations of the *Prdm9* gene (termed alleles), many of which are predicted to bind a unique DNA sequence. This high frequency of variation results in many individuals having two different copies of *Prdm9*, and several lines of evidence indicate that alleles compete to initiate recombination. In seeking to understand the mechanism of this competition we found that *Prdm9* activity is sensitive to the number of gene copies present, suggesting that availability of this protein is a limiting factor during recombination. Moreover, we found that variant forms of PRDM9 protein can physically interact suggesting that when this happens one variant can influence which hotspots will become activated. Genetic crosses in mice support these observations; the presence of a dominant *Prdm9* allele can completely suppress recombination at some locations. We conclude that allele-dominance of PRDM9 is a consequence of protein-protein interaction and competition for DNA binding in a limited pool of molecules, thus shaping the recombination landscape in natural populations.

## Introduction

Genetic recombination in mammals is restricted to hotspots: short, 1–2 kb-long sites scattered throughout the genome [1,2]. With the exception of canids [3,4], their locations in mammals are determined by the sequence-specific DNA binding protein, PRDM9 (MGI:2384854) [5,6,7]. PRDM9 initiates recombination by binding DNA at hotspots where it locally trimethylates histone H3 at lysine 4 (H3K4me3) using a conserved PR/SET domain [8,9,10,11]. This signals the correct locations of programmed meiotic double-strand breaks (DSB) that are required for the physical exchange of material between homologous chromatids during meiosis and the eventual formation of genetic crossovers and noncrossovers [9,10,12].

*Prdm9* function is essential for meiosis; null alleles lead to sterility in both sexes of mice [13], and point mutations in *PRDM9* are found in azoospermic human patients [14,15]. In addition, *Prdm9* is a key player in evolution by creating hybrid sterility. Male intersubspecific F1 hybrid mice that are heterozygous for particular *Prdm9* alleles and carry the *M*. *m*. *musculus*-derived chromosome (Chr) X are infertile, thus creating postmating reproductive barriers that contribute to incipient speciation [16].

*Prdm9/PRDM9* is highly polymorphic, both within and between mammalian species. This includes humans [5,6,7,17,18,19], mice [5,7,9,20], chimps [21,22,23], cattle [24], and equids [25], which all harbor diverse alleles of *Prdm9*. Most of the naturally occurring sequence polymorphisms in *Prdm9* change the identity of the amino acids contacting DNA and/or the number and arrangement of individual fingers in the DNA-binding zinc-finger domains. This allows PRDM9 variants to target a large number of DNA sequences, thereby expanding the distribution of recombination sites.

Three laboratories simultaneously came to the identification of PRDM9 as the key protein determining the location of mammalian hotspots [5,6,7]. In our case, we identified hotspots in genetic crosses between C57BL/6J (B6) and CAST/EiJ (CAST) mice whose activation depended on a *trans*-acting factor [26]. Genetic mapping identified the key factor as the CAST allele of *Prdm9* [7]. Importantly, the same experiments also identified hotspots whose activities were quantitatively reduced rather than activated by the presence of CAST alleles, and others whose activities were completely suppressed. Similar variation in recombination rates has been observed at human hotspots depending on the identities and combinations of *PRDM9* alleles present [17,18,27].

These observations coincide with previous evidence that *Prdm9* alleles in heterozygous individuals do not show simple additive behavior. In both humans [28,29] and mice [10,30] there is allelic dominance in which a predominance of hotspots in heterozygotes are activated by one of the alleles present. This phenomenon is of considerable biological importance given the extensive polymorphism of *Prdm9* and that heterozygotes represent a considerable majority of some natural populations.

Together, the available evidence indicates a complex regulation of hotspot activity in heterozygous individuals. However, little is known of the specific mechanisms and molecular players involved in hotspot suppression and the observed competition between *Prdm9* alleles. Here we report that both of these observations are the functional consequence of a direct interaction between PRDM9 protein variants in a limited pool of PRDM9 molecules in meiotic cells. Using genetic strategies, we now show that, while *Prdm9* is required for activation of hotspots, it is also the *trans*-acting factor responsible for the quantitative modulation of recombination rate and allelic dominance in heterozygous mice. In cell cultures, we show that PRDM9 protein variants form both homo- and heteromeric complexes, and that heteromeric complexes bind and trimethylate nucleosomes at hotspot DNA sequences. We find that *Prdm9* function is dosage-sensitive; in heterozygous male *Prdm9* ^+/-^ null mice, where *Prdm9* is present in a single copy, the numbers and activity of PRDM9-defined H3K4me3 hotspots are reduced and animals have increased abnormalities in meiotic prophase I. In addition, replacing the null allele with one from a different mouse subspecies is sufficient to fully suppress recombination at some hotspots, suggesting direct interaction between protein variants.

Taken together, the data point to a model in which quantitative activity at recombination hotspots is partially controlled through PRDM9 occupancy at hotspots, which in turn is dependent on both the number of PRDM9 molecules available in meiotic cells and the DNA-binding affinity of each allele. Our data suggests that in heterozygous individuals, PRDM9 forms heteromers that preferentially bind to and activate recombination at the stronger allele’s hotspots, thereby suppressing recombination at hotspots otherwise activated by the weaker allele.

## Results

### QTL analysis implicates *Prdm9* in controlling the recombination rate of hotspot *Pbx1*

To determine factors regulating recombination rate, we first focused on the hotspot *Pbx1*, as evidence indicates that genetic background has a strong effect on the recombination rate at this hotspot in mice [26]. *Pbx1* has a sex-averaged recombination rate of 2.38 cM in the B6 background, but shows a significant 5.8-fold down-regulation when CAST alleles are introduced in (B6xCAST)F1 hybrids (0.41 cM, Fisher’s exact test *p* < 10^−4^) [31]. To genetically map factors controlling the quantitative activity of *Pbx1* we used N2 and F2 mapping crosses (S1A Fig) that allowed us to detect the influence of dominant, recessive or additive alleles [26]. We collected 49 N2 and 75 F2 males heterozygous for B6/CAST on distal 100 Mb on Chr 1, the region containing the *Pbx1* hotspot, genotyped them at 165 markers spaced across the genome, and isolated their sperm DNA to measure the recombination rate at *Pbx1*. Crossing over at *Pbx1* was determined using a DNA sequencing assay that takes advantage of SNPs located on either side of the hotspot and counts the number of recombinant and parental molecules in sperm samples (S1B Fig). Comparing the frequencies of parental and recombinant molecules from many thousands of individual sperm (each representing a potential offspring) provided a measure of recombination rates at *Pbx1* in individual male mice.

**Fig 1:**
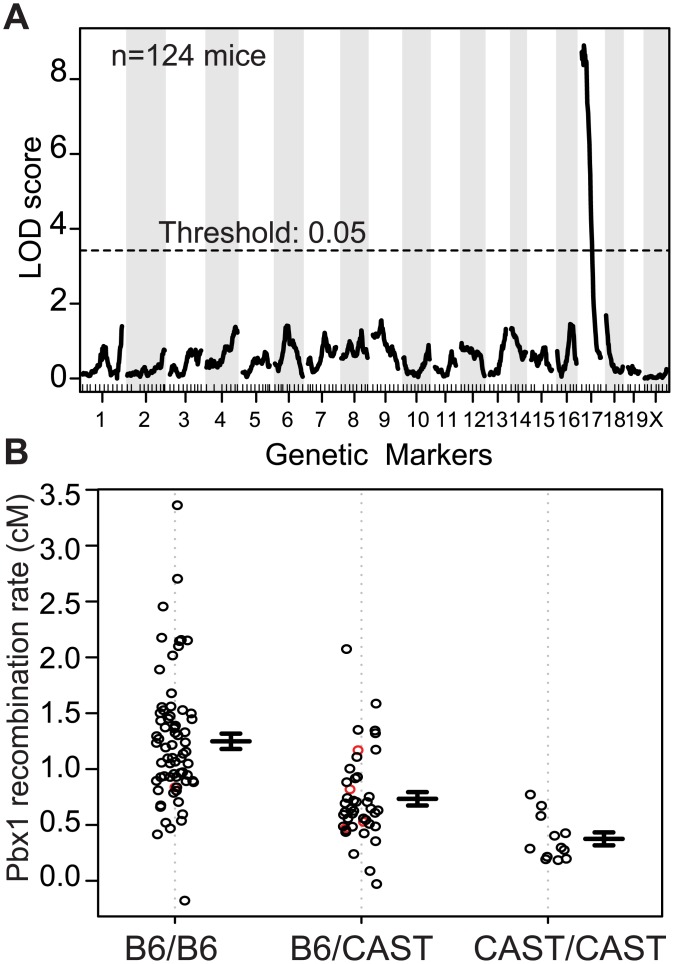
A QTL on proximal Chr 17 influences the recombination rate at *Pbx1*. (A) Manhattan plot showing the result of a single QTL scan from data pooled from N2 and F2 crosses (LOD score = 8.9, threshold determined by permutation). (B) Effect plot showing recombination rate at *Pbx1* from individual mice based on the genotype at the QTL on proximal Chr 17 (red circles, imputed genotypes; error bars, standard error).

Using the recombination rate at *Pbx1* as our phenotype, genome scans performed on individual crosses, and pooled data from both crosses, resulted in a single significant QTL peak on proximal Chr 17 (Figs 1A, S2A and S2B). The 1.5-LOD support interval for this QTL is from ~4–30 Mb along Chr 17, with the approximate QTL located around 14 Mb. Mice homozygous for B6 at Chr 17 had the highest rate of recombination; heterozygous mice had an intermediate level of recombination, and mice homozygous for CAST had the lowest (Fig 1B). This pattern suggests an additive effect of the QTL dependent on the B6 haplotype on Chr 17.

The position of the QTL on Chr 17 implicates *Prdm9* in regulating recombination at the Pbx1 hotspot. *Prdm9* is located on Chr 17 at 15.5 Mb, is currently the only known recombination regulator locus in mice, and B6 and CAST mice carry two different *Prdm9* alleles (*Prdm9* ^*Dom2*^ and *Prdm9* ^*Cst*^ respectively) [7,26]. Furthermore, the PRDM9^Dom2^ protein variant, found in B6 mice where *Pbx1* is active, shows binding to the *Pbx1* DNA sequence in vitro and regulates H3K4me3 level in the surrounding region in vivo [31]. The QTL analysis above suggests that *Prdm9* is also a modifier of recombination rate at *Pbx1*; two copies of *Prdm9* ^*Dom2*^ result in a higher recombination rate at *Pbx1* compared to one copy. The apparent low recombination rate in homozygous CAST at the QTL locus mice is largely a measure of the frequency of false-recombinants (see methods). To test for the presence of additional modifiers of hotspot activity, we conditioned on the identity of the *Prdm9* allele present by selecting the set of N2 and F2 mice that were homozygous *Prdm9* ^*Dom2/Dom2*^ and performed an additional genome scan on these mice alone; however, we did not detect any other significant QTLs (S2C Fig).

### Expression of *PRDM9* in HEK293 cells leads to allele-specific H3K4me3 at hotspots

The genetic evidence above suggests that PRDM9 activation of hotspots is sensitive to *Prdm9* dosage, indicating that PRDM9 is limiting in meiotic cells, or sensitive to competition between alleles. A plausible molecular mechanism by which two alleles can directly influence each other is through their physical interaction [28,29]. To test if this is the case and PRDM9 interacts with itself, we cloned both the human *PRDM9* ^*A*^ allele, the primary allele found in humans from European ancestry, and the *PRDM9* ^*C*^ allele, more prevalent in populations with African ancestry [7,18], for expression in cultured human HEK293 cells similar to previous reports [32,33].

**Fig 2:**
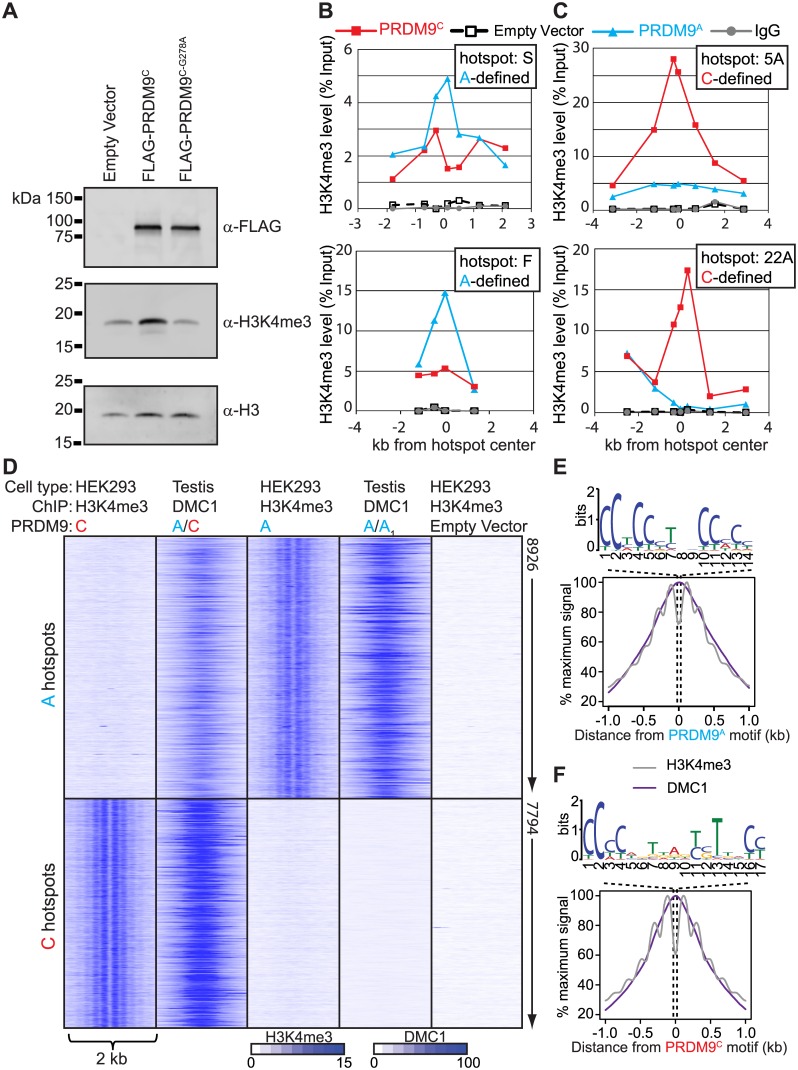
Expression of *PRDM9* in HEK293 cells recapitulates allele-specific hotspot activation in vivo. (A) Western blot showing that expression of PRDM9 in HEK293 cells increases global H3K4me3 levels dependent on the PR/SET domain. (B) and (C) qPCR was performed on enriched DNA from ChIP against H3K4me3 at various intervals across the hotspots indicated by the position of the symbols after expression of PRDM9 protein variants. (B) PRDM9^A^-defined recombination hotspots S and F have increased H3K4me3 after expression of *PRDM9* ^*A*^. (C) PRDM9^C^-defined recombination hotpots 5A and 22A are enriched for H3K4me3 after expression of *PRDM9* ^*C*^. (D) Expression of PRDM9 in HEK293 cells results in allele-specific H3K4me3 at in vivo DSB hotspots. Heat map of H3K4me3 ChIP-seq (HEK293 cells) and DMC1 SSDS (men) signals for a 2 kb window centered on PRDM9 motifs. DMC1 SSDS data from [28]. (E) Aggregate plot of H3K4me3 (grey) and DMC1 (purple) signals from A-hotspots after expression of *PRDM9* ^*A*^. PRDM9^A^ motif derived from the hotspot nucleosome-depleted regions is shown above. (F) Similar to *E* except showing the result for PRDM9^C^.

Expressing *PRDM9* in cultured HEK293 cells resulted in a significant increase in total H3K4me3 levels, which depended on the conserved PR/SET methyltransferase domain present in PRDM9 (Fig 2A), similar to results previously described [13,33]. In order to test if PRDM9 retains DNA-binding specificity in HEK293 cells we expressed *PRDM9* ^*A*^, *PRDM9* ^*C*^, or empty vector and performed ChIP for H3K4me3. Several human hotspots have previously been characterized as being responsive to either PRDM9^A^ (for example hotspots S and F) or PRDM9^C^ (hotspots 5A and 22A) by measuring recombination in pooled sperm samples [17,18]. We found that expression of either *PRDM9* ^*A*^ or *PRDM9* ^*C*^ in HEK293 cells resulted in increased H3K4me3 levels at the center of these hotspots in an allele-specific manner as measured by qPCR (Fig 2B and 2C).

To identify genome-wide PRDM9-defined H3K4me3 sites we used ChIP DNA for deep sequencing, for each allele, and compared these H3K4me3 maps to the recently published genome-wide position of meiotic DSBs identified by chromatin immunoprecipitation of the meiotic recombinase DMC1 from men (S3 Fig and S1 Table, DMC1 SSDS data available at GEO: GSE59836) [28]. DSB hotspots were classified as PRDM9^C^-defined if they were uniquely identified in the DMC1 SSDS data from the heterozygous A/C individual but not found in the homozygous A/A_1_ individual (S3B Fig). For both alleles, approximately one-third of unique allele-specific H3K4me3 sites identified here in HEK293 cells overlap with DSB hotspots identified in testis (S3C and S3D Fig). To visualize allele-specificity, heat maps were generated for each H3K4me3 ChIP and DMC1 ChIP data set at shared hotspots by aligning the position of identified PRDM9 motifs (Fig 2D). H3K4me3 signal at PRDM9^C^-defined DSB hotspots was increased only after expression of *PRDM9* ^*C*^ but not after expression of *PRDM9* ^*A*^ or in empty vector controls. Similarly, expression of *PRDM9* ^*A*^ in HEK293 cells resulted in increased H3K4me3 only at PRDM9^A^-defined hotspots. H3K4me3 signal is readily detected at promoter regions, including empty vector control, highlighting the PRDM9-defined H3K4me3 at hotspots (S4 Fig). For both *PRDM9* alleles, H3K4me3 modified nucleosomes are organized in a symmetrical pattern around a central PRDM9 sequence motif as previously seen in mouse germ cells [9], near the maximum signal of DSB intensity found from testis (Fig 2E and 2F). These data show that ectopically expressed PRDM9 can bind and modify chromatin at hotspot sequences in somatic cells in an allele-specific manner.

### PRDM9 can form heteromeric complexes at hotspots

**Fig 3:**
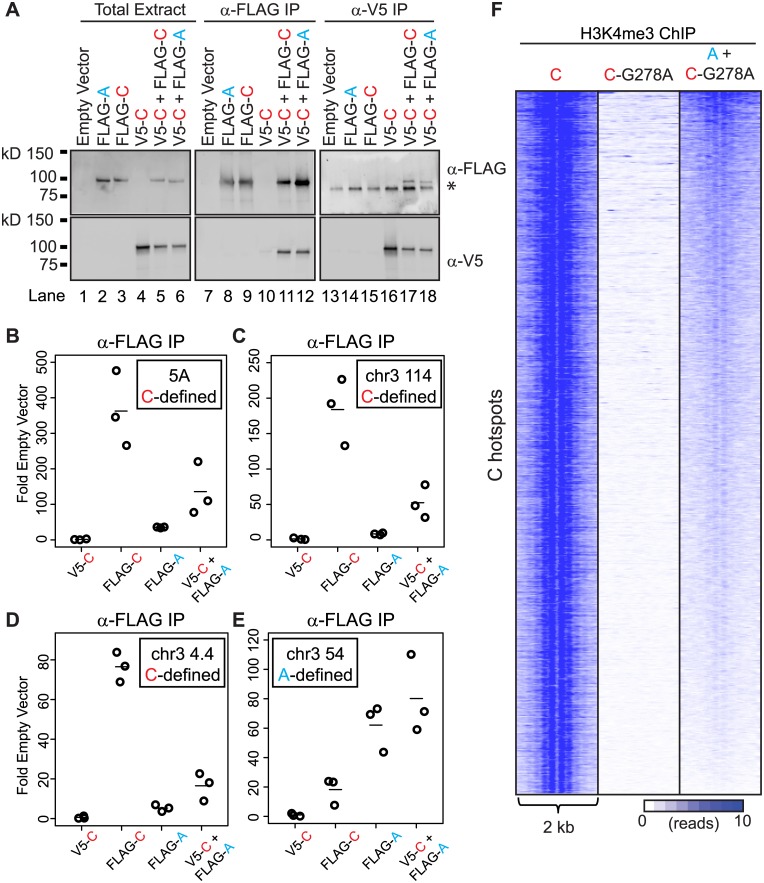
PRDM9 can form heteromeric complexes at hotspots. (A) PRDM9 protein variants can form homo- and heteromeric complexes. Western blot of various tagged versions of human PRDM9 detected either with anti-FLAG or anti-V5 antibodies (asterisk—non-specific band). V5-PRDM9^C^ (V5-C) is found in association with FLAG-PRDM9^A^ (FLAG-A) or FLAG-PRDM9^C^ (FLAG-C) after FLAG or V5 IP. (B) PRDM9^A^ is bound to the PRDM9^C^-activated hotspot 5A when co-expressed with PRDM9^C^. Tagged *PRDM9* alleles, indicated on the x-axis, were transfected into HEK293 cells and subsequently subjected to ChIP against FLAG antibody; qPCR was performed on ChIP DNA samples at the center of the 5A hotspot (circles—individual biological replicates, lines—mean). (C) and (D) Similar to B showing ChIP results for PRDM9^C^-activated hotspots at Chr 3 114 Mb and Chr 3 4 Mb respectively. (E) PRDM9^A^ can bind A-hotspots. Similar to B-D showing ChIP results for PRDM9^A^-activated hotspot at Chr 3 54 Mb. (F) PRDM9^A^ can trimethylate C hotspots when in complex with PRDM9^C^. Heat map of H3K4me3 ChIP-seq read counts from HEK293 cells for a 2 kb window surrounding PRDM9^C^ hotspots from Fig 2D.

To examine if PRDM9 can interact with itself, we assessed interaction between these two human alleles in HEK293 cells. Both alleles were cloned to contain either an N-terminal FLAG or N-terminal V5 epitope tag to facilitate detection and allow discrimination of protein variants. Both the FLAG- and V5-tagged versions of each *PRDM9* allele were expressed, either separately or together, in HEK293 cells (Fig 3A). Immunoprecipitation using the FLAG monoclonal antibody directed against FLAG-PRDM9^A^ or FLAG-PRDM9^C^ showed an enrichment for the V5-tagged PRDM9^C^ only when the two proteins were co-expressed (Fig 3A, lanes 11 and 12). Likewise, reciprocal immunoprecipitation with V5-PRDM9^C^ displayed the same result, as it enriched for both FLAG-PRDM9 protein variants (Fig 3A, lanes 17 and 18). These data show that PRDM9 can form both homo- and heteromeric protein complexes when co-expressed.

A macromolecular complex containing two PRDM9 protein variants would have two distinct zinc-finger arrays, with the potential capability to bind two motifs. This predicts that in cells expressing two *PRDM9* alleles, the non-activating protein variant might be found at hotspots at which it does not typically bind. For example, human PRDM9^A^ would be found at a C-defined hotspot only when in a heteromeric complex with PRDM9^C^. To test for the presence of heteromeric complexes at hotspots, we expressed V5-PRDM9^C^, FLAG-PRDM9^C^, and FLAG-PRDM9^A^ alone, or co-expressed V5-PRDM9^C^ and FLAG-PRDM9^A^ together, and tested for their presence at several C- and A-defined hotspots using ChIP (Figs 3B–3E and S5). As expected, expression of FLAG-tagged PRDM9^C^ (FLAG-C) resulted in enrichment for DNA at C-hotspots following immunoprecipitation using anti-FLAG antibody (Fig 3B, 3C and 3D); while expression of FLAG-PRDM9^A^ (FLAG-A) did not. In addition, using anti-FLAG antibody there was no enrichment for C-hotspots after expression of V5-PRDM9^C^ (V5-C), showing antibody specificity. The lack of PRDM9^A^ signal at C-defined hotspots is not due to inactivity of the protein variant, as PRDM9^A^ can readily bind to an A-hotspot (Fig 3E). Importantly, there were increases in enrichment at C-defined hotspots when co-expressing FLAG-PRDM9^A^ along with V5-PRDM9^C^ (V5-C + FLAG-A) compared to either protein expressed alone (Fig 3B, 3C and 3D). Thus PRDM9^A^, which does not bind to or modify C-defined hotspots alone, is nevertheless found at C-hotspots when co-expressed with PRDM9^C^, a situation potentially similar to heterozygous individuals.

Given that in heteromeric complexes at least two PRDM9 molecules can be found at hotspot sequences, we wanted to test if the protein variant that does not bind DNA can still catalytically function to modify hotspot nucleosomes. To do so we expressed the catalytically-dead FLAG-PRDM9^C-G278A^ alone or co-expressed FLAG-PRDM9^C-G278A^ with V5-PRDM9^A^ and performed ChIP for H3K4me3 (Fig 3F). As expected, expression of FLAG-PRDM9^C-G278A^ alone did not lead to H3K4me3 at C-hotspots. However, when co-expressed with a functional V5-PRDM9^A^ protein variant, C-defined hotspots had a clear, albeit weak, H3K4me3 signal and organized the nucleosome pattern at these hotspots. These data show that bringing together a functional PR/SET domain of one allele, with the zinc-finger DNA-binding domain of a different allele, is sufficient to mark hotspots.

Together, these data provide strong evidence for the formation of functional heteromeric complexes that can bind and modify hotspots.

### B6-*Prdm9* ^*Dom2/-*^ males have reduced H3K4me3 hotspots and display mild meiotic arrest compared to B6

The above cell culture data show that two PRDM9 variants can be found in the same protein complex bound at hotspot DNA sequences. If PRDM9 activity is limiting in meiotic cells, the two protein variants might directly compete for hotspot activation in heterozygous individuals by influencing which allele’s hotspots the heteromeric complexes bind. To investigate if PRDM9 activity is limiting in meiotic cells we next characterized mice made heterozygous null at *Prdm9*.

**Fig 4:**
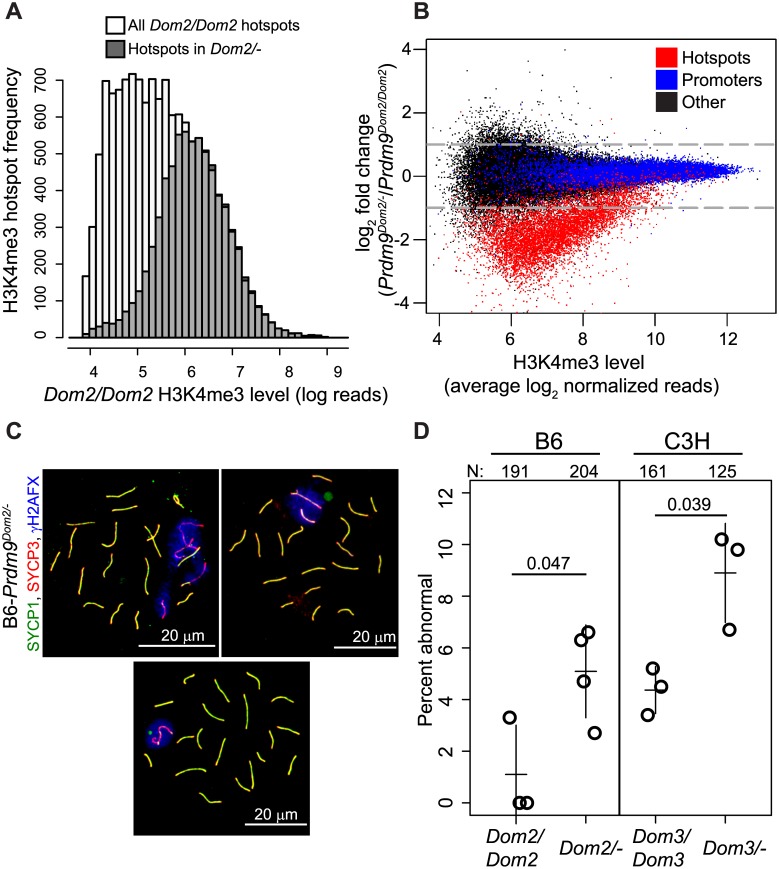
*Prdm9* activity is dosage-sensitive for H3K4me3 levels and partially haploinsufficient for male meiotic progress in vivo. (A) Hotspots found in heterozygous null mice (B6-*Prdm9* ^*Dom2/-*^) correspond to those hotspots with the highest level of H3K4me3 in B6 homozygous mice (B6-*Prdm9* ^*Dom2/Dom2*^). Distribution of H3K4me3 level at hotspots in *Prdm9* ^*Dom2*^ homozygous males (white—H3K4me3 level for all B6 hotspots, grey—H3K4me3 level in B6 mice for hotspots found in *Prdm9* ^*Dom2/-*^ heterozygous males). (B) H3K4me3 activity at hotspots is reduced in *Prdm9* ^*Dom2/-*^ heterozygous mice. MA-plot of H3K4me3 reads from two biological replicates for *Prdm9* ^*Dom2/Dom2*^ and *Prdm9* ^*Dom2/-*^ animals (red—PRDM9-defined H3K4me3 hotspots, n = 7,379; blue—H3K4me3 found at promoters, n = 14,602; black—H3K4me3 found at other functional elements, n = 44,463). (C) Examples of pachytene nuclei from B6-*Prdm9* ^*Dom2/-*^ males obtained by indirect immunofluorescence labeling. Overlapping SYCP1 and SYCP3 serve as markers for synapsis (yellow). ƴH2AFX is a marker for DSBs and should be restricted to the sex body at pachytene stage. Upper left—no sex body and multiple asynapsed autosomes evident by persistence of ƴH2AFX staining; upper right—asynapsed autosome included in the sex body; lower—normal pachynema. (D) The relative number of aberrant pachynemas increases in male mice heterozygous null for two *Prdm9 M*. *m*. *domesticus* alleles (N—total number of nuclei counted; circles—individual male mice; lines—mean ± standard deviation; p-value—Welsch’s t-test).

To test the effect of lowered *Prdm9* dosage on hotspots in vivo, we measured genome-wide H3K4me3 levels in male germ cells from mice heterozygous for the targeted null allele *Prdm9* ^*tm1Ymat*^ (B6-*Prdm9* ^*Dom2/-*^) and compared them to those from homozygous B6 (*Prdm9* ^*Dom2/Dom2*^). B6-*Prdm9* ^*Dom2/-*^ males have reduced level of PRDM9 protein compared to homozygous littermates [30,34], suggesting reduced availability of the catalytic domain. Using H3K4me3 ChIP-seq we identified approximately half the number of detectable H3K4me3 hotspots seen in mice with two copies of *Prdm9* ^*Dom2*^. Among 97,117 total H3K4me3 peaks identified in B6-*Prdm9* ^*Dom2/-*^ germ cells, 9,707 were associated with PRDM9^Dom2^-defined H3K4me3 hotspots, the remainder being associated with promoters and other functional elements. This is in contrast to nearly twice as many H3K4me3 hotspots previously measured in *Prdm9* ^*Dom2/Dom2*^ mice (18,849) [9]. The PRDM9-defined H3K4me3 hotspots identified in the heterozygous null mice correspond to those with the highest level of H3K4me3 in homozygous B6 (Fig 4A).

Next, we compared the relative activity (normalized read counts) of H3K4me3 hotspots present in both B6 and B6-*Prdm9* ^*Dom2/-*^ heterozygous null males. As a class, PRDM9^Dom2^-defined H3K4me3 hotspots have one-third of the level of H3K4me3 in heterozygous null mice compared to B6 (Fig 4B). This reduction in H3K4me3 is also sensitive to the intrinsic strength of the hotspot (as measure by average H3K4me3 level); the weaker the hotspot is (lower average H3K4me3), the greater the fold difference in H3K4me3 levels between B6-*Prdm9* ^*Dom2/-*^ and B6 mice. Importantly, other PRDM9-independent H3K4me3 sites, such as gene promoters, are not affected by *Prdm9* copy number (Fig 4B, blue points). Together, the H3K4me3 ChIP-seq data show that B6-*Prdm9* ^*Dom2/-*^ male mice have about half the number H3K4me3 hotspots compared to B6 mice, and those that are present have reduced levels of H3K4me3, with the greatest reductions seen at the weakest hotspots.

Male mice that are homozygous null for *Prdm9* have a complete meiotic arrest [13,35,36,37]. However, there is conflicting evidence on the effect of removing one allele of *Prdm9* on fertility and meiotic progress. Heterozygous male mice for the targeted null mutation *Prdm9* ^*tm1Ymat*^ have testes weights and sperm counts similar to wild-type males when on a mixed (129*B6) [13] or B6 [36] background. Males heterozygous for another allele, *Prdm9* ^*M1045Lja*^, that expresses a truncated protein [34] displayed lower testes weight, reduced number of spermatids, and azoospermia [37]. To determine the effect of heterozygosity of the *Prdm9* ^*tm1Ymat*^ allele in the B6 background (B6-*Prdm9* ^*Dom2/-*^) on meiotic progress and fertility, we used indirect immunofluorescence labeling of spread adult testicular cells to detect meiotic arrest. Compared to homozygous *Prdm9* ^*Dom2/Dom2*^ B6 littermate controls, B6-*Prdm9* ^*Dom2/-*^ males displayed a mild, but significantly increased fraction of abnormal pachytene stage cells, either completely lacking or having an abnormal sex body (Fig 4C and 4D). This increased number of abnormal pachytene spermatocytes was also seen in heterozygotes when using a different *M*.*m*. *domesticus* allele, *Prdm9* ^*Dom3*^, on a C3H/HeN genetic background (Fig 4D). To assess the effect of lowered *Prdm9* dosage on overall fertility, we crossed heterozygous null (B6-*Prdm9* ^*Dom2/-*^) males to homozygous B6 (*Prdm9* ^*Dom2/Dom2*^) females. The B6-*Prdm9* ^*Dom2/-*^ males produced fewer offspring compared to B6 controls (4.3±1.4 versus 6.2±1.0 per female per month, p = 0.01, Welsch’s t-test) and needed on average 7.4 more days to sire their first litter (p = 0.01, Welsch’s t-test). Thus *Prdm9* is partially haploinsufficient for meiotic progress and fertility.

In total, these data comparing *Prdm9* ^*+/-*^ heterozygous mice to homozygous mice demonstrate that PRDM9 function is dosage-sensitive.

### The number of double-strand breaks is similar between B6 and B6-*Prdm9* ^*Dom2/-*^ male mice

Because the number of H3K4me3 hotspots decreased with lowered *Prdm9* dosage, and B6-*Prdm9* ^*Dom2/-*^ males have increased abnormal pachytene stage cells, we wanted to test if meiotic DSBs are reduced in B6-*Prdm9* ^*Dom2/-*^ males. To accomplish this, meiotic DSBs were counted in early zygonema using indirect immunofluorescence microscopy with a mix of antibodies directed against DSB-repair proteins RAD51 and DMC1 on staged surface-spread testicular nuclei (S6 Fig). The B6-*Prdm9* ^*Dom2/-*^ males displayed 189±18 (mean ± standard deviation) DSBs per cell and their B6-*Prdm9* ^*Dom2/Dom2*^ littermates 202±29 DSBs per cell; this difference was not significant (p = 0.12, Welsch´s t-test), confirming a previous report of no reduction using different combinations of *Prdm9* alleles [34].

### Suppression of hotspot activity goes beyond *Prdm9* dosage

**Fig 5:**
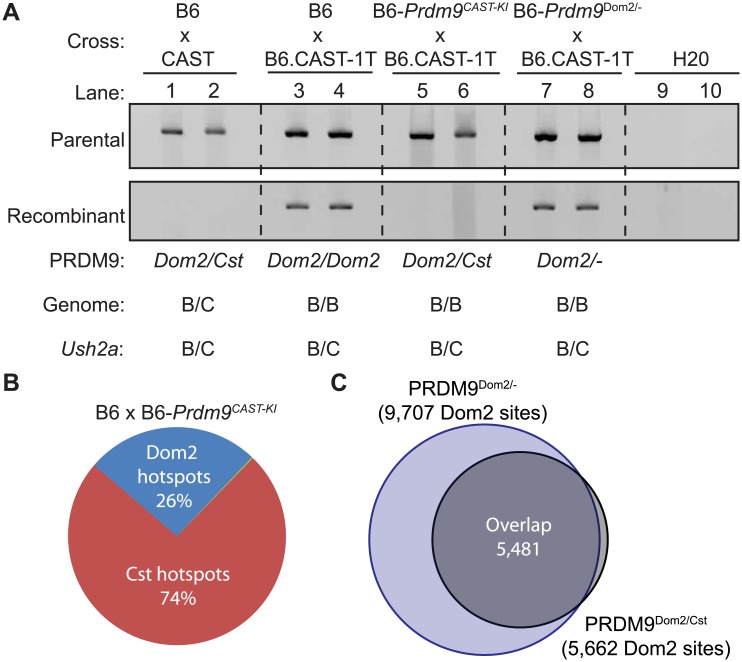
*Prdm9* ^*Dom2*^ is suppressed by competition between alleles. (A) PRDM9^Cst^ can suppress PRDM9^Dom2^-directed recombination at *Ush2a*. To detect either parental (top) or recombinant (bottom) molecules, nested PCR was performed on DNA from pooled sperm samples collected from F1 hybrids from the indicated crosses (two representative biological replicates are shown for each genotype). Recombinant molecules are only detected in progeny homozygous or heterozygous null for *Prdm9* ^*Dom2*^. (B) Proportions of H3K4me3 hotspots in (B6xKI)F1 hybrids due to either PRDM9^Cst^ or PRDM9^Dom2^. (C) PRDM9^Dom2^ hotspots in (B6xKI)F1 hybrids are the same hotspots found in *Prdm9* ^*Dom2/-*^ heterozygous mice.

The combined evidence suggests that PRDM9 activity is limiting in meiotic cells and that PRDM9 variants can self-interact. Together these data suggest that, if heteromeric complexes exist in meiotic cells, the two variants might compete for DNA binding and activation of hotspots in heterozygous individuals. Recombination at some hotspots in *Prdm9* ^*Dom2/Cst*^ heterozygous mice are completely suppressed when CAST alleles are introduced in *trans* [26]. One such example is the PRDM9^Dom2^-defined hotspot *Ush2a* (genomic position: Chr 1 190,124,179–190,127,477 Mb). By genotyping progeny from crosses [26], we found that the sex-averaged recombination rate at *Ush2a* is 0.61 cM in the B6 background and completely suppressed when CAST alleles are present (Fisher’s exact test *p* < 10^−4^). Nested allele-specific PCR, using primers to amplify either parental or recombinant molecules from pooled sperm, confirms the genetic cross data (Fig 5A). Crossovers at *Ush2a* are detected in sperm DNA from (B6 x B6.CAST-1T)F1 hybrids that are heterozygous B6/CAST at the hotspot on distal Chr1 and otherwise homozygous B6/B6 (Fig 5A, lanes 3 and 4), but fully suppressed in sperm DNA from (B6 x CAST)F1 hybrids that are heterozygous B6/CAST across all of the genome (Fig 5A, lanes 1 and 2).

To test if suppression of recombination is due to reduced *Prdm9* ^*Dom2*^ dosage, competition between PRDM9^Dom2^ and PRDM9^Cst^, or the action of a novel regulatory factor, we compared recombination at *Ush2a* in sperm DNA from co-isogenic mice that are either heterozygous *Prdm9* ^*Dom2/Cst*^ or heterozygous *Prdm9* ^*Dom2/-*^, heterozygous B6/CAST on distal Chr 1 (to allow detection of crossing over at *Ush2a*), and uniformly B6/B6 over the rest of the genome. We did so by using appropriate progeny from two crosses: B6-*Prdm9* ^*CAST-KI*^ (KI), a co-isogenic strain in which the *Prdm9* ^*Dom2*^ allele has been replaced by the *Prdm9* ^*Cst*^ allele from CAST mice [9], crossed to B6.CAST-1T (KI x CAST-1T), and B6-*Prdm9* ^Dom2/-^ crossed to B6.CAST-1T (KO het x CAST-1T). Importantly, similar to (B6 x CAST)F1 hybrid males, recombination at *Ush2a* is suppressed in (KI x CAST-1T)F1 hybrid males, where the only difference is the presence of *Prdm9* ^*Cst*^ in one copy (Fig 5A, lanes 5 and 6). However, recombination persists in (KO het x CAST-1T)F1 hybrid males, which only have one allele of *Prdm9* ^*Dom2*^ (Fig 5A, lanes 7 and 8). Moreover, the rate of recombination at *Ush2a* is similar in B6 mice with two doses of *Prdm9* ^*Dom2*^ and heterozygous B6-*Prdm9* ^*Dom2/-*^ mice with one dose (S2 Table). These data show that the *Prdm9* ^*Cst*^ allele alone is sufficient to directly suppress the activity of the *Prdm9* ^*Dom2*^ allele at the *Ush2a* hotspot.

### *Prdm9* alleles compete for hotspot activation in heterozygous animals

We next tested the extent to which *Prdm9* ^*Cst*^ can influence *Prdm9* ^*Dom2*^ activity on a genome-wide basis. Previous reports found that, when tested for either H3K4me3 initiation sites or DMC1 DSB sites, the number of PRDM9^Dom2^-defined hotspots represent much less than the predicted 50% of all hotspots in F1 hybrids carrying two different *Prdm9* alleles, indicating some form of competition between alleles [9,10]. In progeny from both B6xCAST and CASTxB6 crosses, the majority (65%) of all hotspots were PRDM9^Cst^-activated [30]. However, interpretations of genome-wide hotspot behavior in traditional F1 hybrids between inbred strains are complicated by the presence of novel hotspots not found in either parent that result from the action of one parents *Prdm9* allele on the genome of the other parent [30], and by the fact that the entire genome is heterozygous, potentially introducing additional *trans* control mechanisms.

To remove these complications and test for competition in a genetically uniform background, we crossed B6 mice to co-isogenic B6-*Prdm9* ^*CAST-KI*^ mice and measured H3K4me3 levels in germ cells of the resulting heterozygous *Prdm9* ^*Dom2/Cst*^ F1 male progeny. The total number of putative PRDM9-defined H3K4me3 hotspots in *Prdm9* ^*Dom2/Cst*^ progeny (n = 21,894, Fig 5B) is less than the sum of the parental strains (n = 18,849 and n = 28,475, for B6 and CAST-KI respectively) [9], similar to previous result for DSBs in crosses involving *Prdm9* ^*Dom2*^ [10], likely reflecting the sensitivity of hotspot numbers to the total amount of PRDM9 protein. In addition, only ~ 26% of H3K4me3 hotspots in these F1 mice are PRDM9^Dom2^-activated, while ~ 74% are PRDM9^Cst^-activated (Fig 5B). The PRDM9^Dom2^-activated H3K4me3 hotspots that are found in the (B6xKI)F1 mice are a subset of the PRDM9^Dom2^ hotspots found in *Prdm9* ^*Dom2/-*^ heterozygous mice (Fig 5C), and are therefore those with the highest activity in B6 mice. Together these data confirm competition between alleles in mice heterozygous for *Prdm9* and may suggest that PRDM9^Cst^ has a greater affinity for its binding sequence.

## Discussion

In both mouse and humans, recombination rates can be influenced by heterozygosity at *Prdm9* [17,18,26]. Here, using mouse genetics, we identified a single QTL influencing the recombination rate at the *Pbx1* hotspot that overlaps with *Prdm9* (Fig 1). The QTL mapping data suggested that *Prdm9* function is more complex than simple activation of hotspots; in particular, that it is dosage-sensitive and subject to competition between alleles in heterozygous individuals. We found that PRDM9 can form homo- and heteromeric complexes, and that these complexes are bound to DNA at hotspots (Fig 3), providing a molecular explanation for competition between alleles in both mouse and humans. Moreover, measuring H3K4me3 levels at hotspots in heterozygous null and homozygous mice confirmed that *Prdm9* is dose-sensitive (Fig 4). Finally, we found that hotspot suppression extends beyond simple dosage of *Prdm9* in heterozygous mice, showing that the PRDM9^Dom2^-activated hotspot *Ush2a* is directly suppressed by the presence of only the *Prdm9* ^*Cst*^ allele (Fig 5).

Our data indicate that *Prdm9* is partially haploinsufficient for mouse fertility on the B6 background. Further phenotypic evidence for *Prdm9* dosage sensitivity comes from genetic studies of hybrid sterility [16,36,38]. Crosses between certain *M*. *m*. *musculus*-derived mice and *M*. *m*. *domesticus* strains carrying *Prdm9* ^*Dom2*^ result in sterile males with the severity of the pachytene-stage arrest in spermatogenesis being dependent on the parental origin of Chr X [39]. Thus, there are complex genetic interactions between PRDM9 protein variants and another locus on Chr X [38,40]. The F1 hybrid male sterility can be rescued by either making the *Prdm9* ^*Dom2*^ allele homozygous; replacing the *Prdm9* ^*Dom2*^ allele with another *M*. *m*. *domesticus* allele; or adding extra copies of *Prdm9* (independent of which *M*. *m*. *domesticus* allele is added); or it can be partially rescued by removing *Prdm9* ^*Dom2*^, creating a heterozygous null *M*. *m*. *musculus* state, these results together further implicate allelic interactions in the hybrid sterility phenomenon [36]. These data, together with our findings on the capacity of PRDM9 to form homo- and heteromeric complexes, indicate that the sterility phenotype is *Prdm9* dosage-sensitive and may be partially explained by incompatibilities of different homo- versus heteromeric PRDM9 complexes.

In the absence of *Prdm9*, meiotic DSBs persist [13], although they are relocated away from recombination hotspots to other *Prdm9*-independent H3K4me3 sites such as those found at promoters and enhancers [10], resulting in complete meiotic arrest. We found that B6-*Prdm9* ^*Dom2/-*^ heterozygous mice have a partial failure in meiotic progression (Fig 4). One possible explanation is that the reduced number of PRDM9-dependent H3K4me3 hotspots in a single cell may lead DSBs to be redirected to other, PRDM9-independent, H3K4me3 sites, which are subsequently not properly repaired, as occurs in the homozygous null mouse [10].

Evidence for competition between *PRDM9* alleles is also seen in humans [17,18,29]. The recombination rate at several hotspots was measured in men carrying various combinations of A-type and C-type *PRDM9* alleles. While men homo- or heterozygous for PRDM9^C^ have similar recombination rates at C hotspots, recombination rates at A hotspots are reduced in heterozygous *PRDM9* ^*A/C*^ men when compared to homozygous *PRDM9* ^*A/A*^ men [29]. In addition, in one heterozygous *PRDM9* ^*A/C*^ man, 56% of the DSBs were due to PRDM9^C^ protein variant, and PRDM9^C^ hotspots were on average stronger than PRDM9^A^ hotspots [28]. Data from these observations, combined with our finding that PRDM9^A^ and PRDM9^C^ can form heteromers and that PRDM9^A^ can be found at C hotspots, support the idea that competition between human *PRDM9* alleles results from PRDM9^C^ being partially-dominant to PRDM9^A^, a relationship similar to that of PRDM9^Cst^ and PRDM9^Dom2^ in mice.

In addition to *Prdm9*, there are 16 orthologous *PRDM* genes in primates and 15 orthologs in rodents, many of which function in multi-protein complexes [41,42]. PRDM proteins are characterized as containing a PR/SET domain, which can catalyze a variety of chromatin modifications, and most also have C-terminal DNA-binding zinc finger domains. Two other PRDM-family proteins, PRDM6 and PRDM2 (also known as Riz1), also form homomeric complexes, in part through interactions involving their PR/SET domains [43,44]. PRDM9 also contains a KRAB domain known to facilitate protein-protein interactions [45]. The mouse and human genomes both contain hundreds of other KRAB-Zinc finger proteins [46], and several are known to form both homo- and heterodimers [47]. Together, these observations suggest that multimer formation may be a common feature of PRDM and KRAB domain containing proteins.

**Fig 6:**
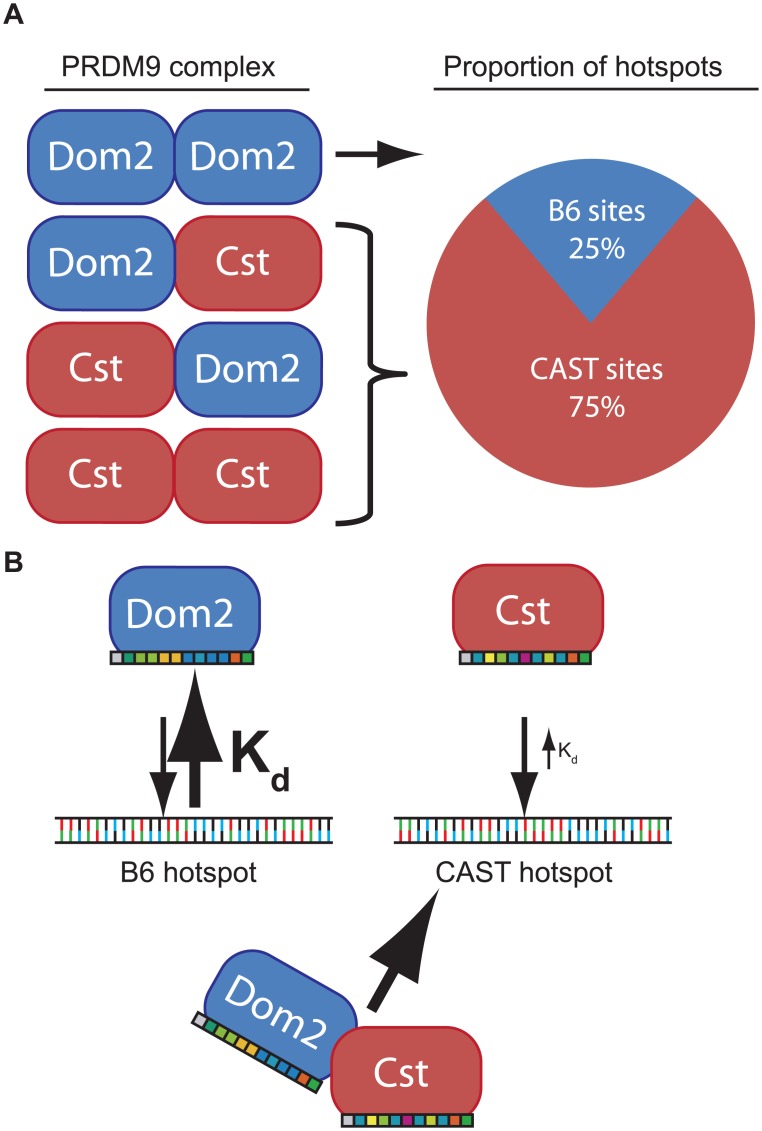
Model of PRDM9 dimerization explains allelic competition and recombination in heterozygotes. (A) In the simple case of a protein dimer heterozygous individuals would be expected to form PRDM9 protein complexes in a 1:2:1 ratio. Because PRDM9^Cst^ is dominant to PRDM9^Dom2^, most heterodimers would activate PRDM9^Cst^ hotspots. (B) Each PRDM9 allele is predicted to bind an allele-specific set of hotspots through allele-specific zinc-finger domains (colored boxes). If intrinsic binding affinity, determined through each zinc-finger array, is different between alleles, a heterodimer would be predicted to be bound more often at hotspots of the allele with the smaller dissociation constant (K_d_).

The phenomenon of dominance among *Prdm9* alleles is most simply explained by assuming that different alleles have different intrinsic DNA binding affinities determined by the allele-specific zinc finger domains. For example, in a heterozygous mouse, such as the F1 offspring of a cross between B6 and CAST mice, putative PRDM9 dimers would consist of PRDM9^Dom2^ homodimers, PRDM9^Dom2^-PRDM9^Cst^ heterodimers, or PRDM9^Cst^ homodimers, in approximate ratios of 1:2:1 (Fig 6). If PRDM9^Cst^ is dominant over PRDM9^Dom2^, as the suppression of *Ush2a* suggests, and overall PRDM9 activity is limiting, as the results from the B6-*Prdm9* ^*Dom2/-*^ studies indicate, PRDM9^Dom2^-PRDM9^Cst^ heterodimers would activate PRDM9^Cst^-defined hotspots more often than PRDM9^Dom2^ hotspots, predicting the 3:1 over-representation of PRDM9^Cst^-defined hotspots that are active in (B6 x KI)F1 hybrids (Figs 5B and 6). The dominance relationship seen between these two alleles is enhanced by the fact that PRDM9^Dom2^ hotspots have undergone greater evolutionary hotspot erosion in B6 mice compared to PRDM9^Cst^-defined hotspots, resulting in PRDM9^Cst^ hotspots having greater binding affinity in the B6 background [9,30]. However, not all allelic pairs show such large bias in hotspot selection. For example, in (WSB x PWD)F1 hybrids, containing two different PRDM9 alleles, 32% of hotspots are defined by the WSB allele and 40% of hotspots are defined by the PWD allele, and the remaining hotspots are unique to the F1 [30]. In any particular combination of alleles, relative dominance will be determined by the intrinsic binding strength of each allele for the hotspots found in that genetic background. This model is supported by the following evidence: *Prdm9* activity is dosage dependent (Fig 4), suggesting a limited molecular activity within meiotic cells, PRDM9 protein variants directly compete for hotspot binding [30], for H3K4me3 activity (Fig 5B), DSBs [10,28], and genetic recombination (Fig 5A) [17,18,29], and finally, PRDM9 can form heteromeric complexes that allow protein variants to directly influence each other (Fig 3).

In general, if the average affinity of a PRDM9 allele for its hotspots is appreciably stronger than that of a different PRDM9 allele for its hotspots, and the two protein variants are found in complex together, this difference in affinity in heterozygotes would create a molecular tug-of-war with the stronger allele winning more often, further diminishing the effective dose of the weaker allele. As a result, in heterozygotes, a complex containing both PRDM9 protein variants would more often be bound at hotspots corresponding to the stronger allele. Given the very high population frequencies of *Prdm9* heterozygotes [17,18,23,28,48,49], these effects can seriously influence patterns of inheritance in some natural populations.

## Materials and Methods

### Mice

The animal care rules used by The Jackson Laboratory and Institute of Molecular Genetics are compatible with the regulations and standards of the U.S. Department of Agriculture, National Institutes of Health, and European Union Council Directive 86/609/EEC and Appendix A of the Council of Europe Convention ETS123. The protocols were approved by the Animal Care and Use Committee of The Jackson Laboratory (Summary #04008) and Committee on the Ethics of Animal Experiments of the Institute of Molecular Genetics (Permit Nos. 137/2009, 61/2013).

C57BL/6J (stock number 000664) and CAST/EiJ (stock number 000928) mice were used. The generation and characterization of the B6.CAST-1T, B6-*Prdm9* ^*CAST-KI*^/Kpgn and B6-*Prdm9* ^tm1Ymat^ strains were described previously [9,13,26,38,50]. The C3H-*Prdm9* ^tm1Ymat^ mice were derived from *Prdm9* ^tm1Ymat^ mice by repeated backcrossing to C3H/HeN resulting in a 98% C3H/C3H background; the differential segment of Chr 17 carrying *Prdm9* was approximately 36 Mbp.

### Indirect immunofluorescence labeling and microscopy

Surface-spread testicular nuclei were prepared using the hypotonic treatment protocol described previously [51] with a few modifications [36,38]. The following antibodies were used: rabbit polyclonal anti-RAD51 (Santa Cruz, sc-8349), anti-DMC1 (Santa Cruz, sc-22768), and anti-SYCP1 (Abcam, ab15087); mouse monoclonal anti-γH2AFX (Upstate, #05–636) and anti-SYCP3 (Santa Cruz, sc-74569); goat anti-Rabbit IgG Alexa Fluor 488 (Molecular Probes, A-11034); and goat anti-Mouse IgG Alexa Fluor 568 (Molecular Probes, A-11031) and IgG Alexa Fluor 647 (Molecular Probes, A-21236). The images were acquired using a Nikon E400 microscope with a DS-QiMc mono-chrome CCD camera (Nikon) and processed in the NIS-Elements program (Nikon). The spread nuclei were staged based on axis development and synaptonemal complex formation.

### Sequencing assay for measuring meiotic recombination and QTL mapping

An important goal for counting recombination at *Pbx1* was to bring distant (1–2 kb) SNPs that define recombination hotspots into close proximity for DNA sequencing in a single molecule, while being able to multiplex DNA from hundreds of animals in one sequencing lane. This was achieved using a series of enzymatic steps designed to reduce false-recombinant molecules and incorporate DNA barcoded primers (S1B Fig). Using this system each molecule sequenced represents a single sperm DNA and therefore a potential recombinant DNA. Epididymal sperm was collected from adult mice and DNA purified using the automated sample handling system Maxwell 16 (Promega) with the Tissue LEV Total RNA Purification Kit (Promega). All mice were genotyped as described previously [26].

*Step 1*: First-round of PCR. DNA primers were design to amplify Pbx1 and contained NotI restriction sites (all primers are listed in S3 Table). PCR reactions for each sample were seeded with ~20,000–25,000 haploid genomes (75 ng total sperm DNA) using 0.25 μl Phusion II enzyme with the HF Buffer (New England Biolabs), 0.8 μM of each primer, 5% DMSO, 0.2 mM dNTPs (NEB) in a total reaction volume of 25 μl. First-round PCR conditions include an initial 98°C 30 second denaturing step followed by 11 cycles of 98°C for 10 seconds, and a 70°C annealing step for 30 seconds followed by 72°C extension step for 45 seconds. The final cycle was followed by 10 minutes at 72°C. *Step 2*: The entire PCR reaction was brought to 50 μl supplemented with 1 μ NotI (NEB) and appropriate restriction buffer and incubated for 60 minutes at 37°C, followed by heat-inactivation at 80°C for 15 minutes. *Step 3*: To facilitate intra-molecular ligation and create circularized DNA, the restriction digests were diluted to 200 μl using 5% polyvinylpyrrolpidone (SIGMA), 20 μl T4 ligase buffer, and 1 μl T4 Ligase (NEB). Ligations were performed at 15°C for 15 minutes. The ligation reactions were treated with Exonuclease I and III and incubated at 37°C for 15 minutes to digest any remaining linear DNA molecules. Exonuclease was heat-inactivated by incubating at 95°C for 2 minutes. DNA was concentrated using standard ethanol precipitation and diluted in 10 mM Tris pH 8.0. *Step 4*: The second round of PCR was performed to generate small DNA molecules amenable to paired-end sequencing. PCR reaction conditions were similar to the first round of PCR in a total reaction volume of 25 μl. Second-round PCR primers were designed to include an 8-bp DNA barcode on the 5’ end in order to allow multiplexing different mouse samples. PCR cycling conditions were also similar to the first round of PCR using 24 cycles. *Step 5*: After the second round of PCR all individual 25 μl reactions were pooled together and concentrated using ethanol precipitation and resuspended in 10 mM Tris pH 8.0. DNA was run on 2% agarose gel for size selection and purification using QIAquick Gel Extraction Kit (Qiagen). The resulting samples were then subject to high throughput DNA sequencing (see below).

Even with the protocol described above, the DNA sequences generated by high-throughput sequencing consistently reported a low rate of recombination in control samples. To measure false-recombination using deep sequencing we mixed equal amounts of spleen DNA prepared from B6 and CAST mice separately prior to the first-round of PCR; this analysis resulted in a false-recombination rate of 0.22 ± 0.05 cM (mean ± standard deviation). Because recombination cannot occur in these control samples, we conclude that the observed chimeric molecules are created from incomplete extension in one PCR cycle synthesizing a DNA molecule that is subsequently used to prime DNA in following rounds of PCR, so called template-switching or ‘jump-PCR’.

QTL analysis was performed using R (http://www.R-project.org/) and the r/qtl package [52]. Single-QTL scans were performed using the scanone function using imputation method. Genome-wide LOD significance thresholds were defined by performing 5,000 permutations.

### Plasmid constructs

The *PRDM9* ^*B*^ allele was purchased from OriGene (Rockville, MD). Oligonucleotide primers were designed to include a 5’ V5 epitope tag and used to amplify the full-length *PRDM9* and cloned into pCEP4 expression vector (Invitrogen) to create pCB09. A 6X-HIS-3X-FLAG tag was inserted in frame replacing the V5-tag using yeast-based homologous recombination [53]. The zinc-finger arrays for both *PRDM9* ^*A*^ and *PRDM9* ^*C*^ were amplified from human genomic DNA [7] and cloned into pBAD-HisC (Invitrogen). These zinc-finger arrays were subcloned into the pCEP4 vectors using restriction enzymes AflII and HindIII (NEB) to create full-length tagged versions of FLAG-PRDM9^C^ (pCB51), V5-PRDM9^C^ (pCB47), and FLAG-PRDM9^A^ (pCB53), and V5-PRDM9^A^ (pCB48) for expression in mammalian cell culture. The V5-PRDM9^C-G278A^ allele was created using QuikChange II site-directed mutagenesis (Agilent Technologies) to change glycine 278 to alanine to create pCB56. All cloning oligonucleotides are listed in S3 Table.

### Cell culture and transfection

HEK293 cells were cultured in DMEM (Gibco, Life Technologies) supplemented with 10% FBS (Gibco) at 37°C and 5% CO_2_. 24 hours prior to transfection, cells were seeded at with 10 ml 2.5·10^5^ cell/ml in 10-cm culture-treated plates. Cells were transfected using X-tremeGene HP transfection reagent (Roche) following manufacturer’s protocol using a ratio of 3:1 reagent to DNA with 10 μg total plasmid DNA.

### Chromatin immunoprecipitation

H3K4me3 ChIP-seq from mouse spermatocytes was performed as previously described [9]. ChIP from HEK293 cell cultures were performed with modifications. After transfection cells were allowed to grow for 48 hours. For H3K4me3 ChIP cells were crosslinked by adding formaldehyde (SIGMA) to a final concentration of 1%, and incubated for 10 minutes. For FLAG-tagged PRDM9 ChIP, cells were crosslinked using freshly prepared paraformaldehyde added to a final concentration of 1% and incubated for 5 minutes. Excess formaldehyde was quenched by adding glycine to a final concentration of 125 mM. The medium was removed and the cells were washed once with phosphate-buffered saline (PBS, SIGMA). The PBS was removed and 2 ml of fresh PBS was added supplemented with protease inhibitor cocktail (SIGMA). The cells were collected by scrapping into a 2-ml Eppendorf tube and pelleted by centrifugation at 5000 x g at 4°C for 5 minutes. The PBS was removed and the cell pellet frozen in liquid nitrogen and stored at -80°C. For H3K4me3 ChIP chromatin isolation, MNase digestion, and immunoprecipitation steps were carried out as previously described for spermatocytes. For FLAG ChIP, chromatin was sheared using sonication and immunoprecipitation performed as described for mouse PRDM9 [30].

### High-throughput sequencing and data processing

Pooled DNA samples from the *Pbx1* recombination assay were prepared for sequencing using the TruSeq DNA PCR-Free Sample Preparation Kit (Illumina) in order to avoid PCR amplification, which could lead to template switching during amplification, in turn leading to false recombinant molecules. After library preparation *Pbx1* DNA was size-selected using the Pippin Prep (Sage Science). DNA from ChIP experiments was prepared for sequencing using NEXTflex ChIP-Seq Kit (Bioo Scientific) for H3K4me3 ChIP from mouse spermatocytes, or Kapa Hyper Prep Kits (Kapa Biosystems) for H3K4me3 ChIP from HEK293 cells without size-selection and 14-cycle PCR amplification. Sequencing for mouse samples was performed at The Jackson Laboratory using the Illumina HiSeq 2000 platform. Sequencing for HEK293 samples was performed at the New York Genome Center using Illumina HiSeq 2500 platform. Base calls were made using CASAVA and mapped to either the mouse genome (mm9) or the human genome (hg19) using BWA [54] with default settings. Custom software was developed to count parental and recombinant molecules, and to de-multiplex individual mice from the *Pbx1* recombination assay. For ChIP-seq, alignment files were filtered to keep only uniquely mapped reads. DMC1 SSDS (DSB) ChIP data was previously described [28] (GEO accession no. GSE59836). Peak calling was performed using MACS (v.1.4.2) [55] using ChIP samples for treatment and, for H3K4me3 ChIP, sequenced input DNA as controls with the following settings:-p 1e-5 –keep-dup = ‘all’. Coverage profiles presented in figures were generated with the UCSC genome browser (settings: mean, smoothing window 5) using bedgraphs generated from MACS after tag-shifting. Motif identification and searching for PRDM9^C^ and PRDM9^A^ allele-specific motifs was performed using the MEME Suite (v. 4.9.0) [56]. To locate hotspot centers for heat maps, for each hotspot with more than one motif instance only the top scoring motif was retained (threshold—p-value < 0.0001). Analysis of H3K4me3 peak differences between B6 and heterozygous B6-*Prdm9* ^*Dom2/-*^ null mice was performed using the R package DiffBind [57].

Heat maps for H3K4me3 ChIP from HEK293 cells and DMC1 ChIP were created using seqMiner [58] for peaks with identified PRDM9 motifs that overlapped both H3K4me3 and DMC1 datasets. For heat maps, tag extension was set at 150 bp for H3K4me3 and 450 bp for DMC1 ChIP, determined by the MACS tag-shifting model, and a wiggle step of 1 bp. Summaries of H3K4me3 ChIP-seq and DMC1 SSDS datasets are presented in S1 Table and S3 Fig. Analysis of peak locations between datasets was performed using bedtools [59].

### Quantitative PCR

Quantitative PCR (qPCR) was performed using Quantifast SYBR Green PCR Kit (Qiagen) on the real-time PCR system MasterCycler ep realplex (Eppendorf). Primers were designed using OligoPerfect primer design software (Life Technologies) with 40–60% GC with a product size of 80–120 bps (all primer sequences are listed in S3 Table). All PCR reactions were set up in technical triplicates with 2 μl of ChIP DNA and 0.5 μM forward and reverse primers. Reactions were run for 40 cycles followed by melting curve analysis, and cycle threshold numbers were determined by automated threshold. All ChIP samples were normalized to purified input DNA controls.

### Western blots and immunoprecipitation

Whole-cell protein was extracted from HEK293 cells using RIPA buffer (SIGMA) supplemented with 1 mM PMSF, 1X protease inhibitor cocktail (SIGMA), 1 mM EDTA, 1 mM DTT, and 1 μl Benzonase (SIGMA). Cells were lysed at 4°C for 30 minutes mixed every 5 minutes. For Histone extraction, cells were first incubated for 30 minutes with rotation in hypotonic lysis buffer (10 mM Tris-HCL, pH 8.0; 1 mM KCl, 1.5 mM MgCl_2_) supplemented with 1 mM PMSF and 1X protease inhibitor cocktail. Nuclei were pelleted by centrifugation at 10,000 x g for 10 minutes at 4°C. Histones were recovered by diluting nuclei in 0.2 N HCl and incubating at 4°C with rotation for 2 hours. Cell lysate was cleared by centrifugation at 10,000 x g for 10 minutes at 4°C. Protein samples were normalized for equal loading using Bradford Reagent (BioRad) and diluted in SDS gel-loading buffer and heat-denatured for 5 minutes at 98°C.

For immunoprecipitation, cleared whole-cell lysate was diluted to 500 μl in RIPA buffer. Magnetic protein-G Dynabeads (Invitrogen) were pre-washed with RIPA and treated with anti-FLAG or anti-V5 antibodies for 20 minutes with rotation at room temperature, and washed again with RIPA. Dynabeads were added to the whole-cell lysates and incubated with rotation at 4°C for 3 hours. Immunocomplexes bound to beads were washed 3 times with 500 μl RIPA buffer and eluted using 2X SDS loading buffer.

For western blotting, protein was loaded into 4–15% Tris-Glycine gels (mini-protean, BioRad) and electrophoresis was carried out at 150 V for 60 minutes. Protein was transferred to nitrocellulose membranes using the iBlot system (Invitrogen) with a 7-minute transfer. Westerns were developed using the SNAP i.d. 2.0 Protein Detection System (Millipore/EMD) with the following antibodies diluted 1:1000: anti-FLAG M2 (SIGMA, F1804), anti-V5 (Invitrogen, R960-25), anti-H3K4me3 (Millipore/EMD, 07–473), anti-H3 (Millipore/EMD, 06–755), and VeriBlot secondary antibody HRP (Abcam, ab131366). Blots were visualized using enhanced chemiluminescent substrate SuperSignal West Femto (Life Technologies) and images digitally captured using a G:BOX gel document system (SYNGENE).

### *Ush2a* recombination assay

Sperm DNA was amplified by two rounds of nested PCR using allele-specific primers in each PCR reaction similar to previously described [7]. The two pairs of primers were orientated in 5’-3’ CAST-B6 combination. The 5’ forward primers were both designed to the CAST haplotype. The 3’ reverse primers were designed as either CAST or B6. Primers were PTO-modified at the last two nucleotides in the 3’ end (primer sequences found in S3 Table). The first-round PCR was performed using 50 ng sperm DNA, 0.25 mM of each dNTP, 0.25 μM of each primer, 1x Titanium Taq PCR buffer, and 0.5 U Titanium DNA Taq Polymerase (Clontech Laboratories Inc). PCR cycling conditions included an initial denaturizing step at 94°C for 5 minutes, then 12 cycles of 94°C for 1 minute, 64°C for 40 seconds, and extension time of 68°C for 3 minutes, followed by a final extension time at 68°C for 10 minutes. The amplified DNA product was diluted 10 times and 2 μl used for the second-round allele-specific PCR. Second-round PCR cycling conditions used an initial denaturing step at 94°C for 5 minutes, then 40 cycles of 94°C for 1 minute, 55°C for 40 seconds, and extension time of 68°C for 3 minutes, followed by a final extension time at 68°C for 10 minutes.

Quantitation of recombination rates was done by determining the number of crossover and parental molecules in the same sample of sperm DNA. PCR amplification was carried out in serial dilutions where the starting amount of DNA was diluted two times in each consecutive reaction. The last positive and the first negative dilution reactions were used to perform 20 PCR reactions each in parallel. The number of negative reactions in each pool determines the number of amplifiable molecules through the Poisson distribution.

### Data access

High-throughput sequencing files and processed data for ChIP-seq experiments associated with this manuscript can be found at Gene Expression Omnibus under accession numbers GSE52628 and GSE67673.

## Supporting Information

S1 Fig Strategy to measure recombination in pooled sperm samples. (A) Genetic strategy for QTL mapping. In the N2 cross, congenic B6.CAST-1T mice, whose distal 100 Mb of Chr 1 was derived from CAST mice, were mated to CAST mice. The F1 hybrid progeny from this cross are heterozygous B6/CAST except for the distal 100 Mb on Chr 1 where they remain homozygous CAST. These F1 mice were backcrossed to B6 mice to collect N2 males, all of which are B6/CAST heterozygous for the distal half of Chr 1, to quantitate crossing over in pooled sperm samples. In the second cross B6 and CAST mice were mated to produce an F2 population, and male offspring that were heterozygous B6/CAST on the distal half of Chr 1 were selected. This panel was inspired by Fig 1. in [26]. (B) Hotspots are defined by recombination between flanking SNPs that can be between 2–3 kb apart. DNA molecules these sizes are not easily sequenced using most current high-throughput sequencing methods. This method was developed in order to bring flanking SNPs within close proximity for sequencing and to allow multiplexing of many individual mice into one sequencing reaction. *Step 1*: PCR was performed with limited cycles using common primers designed to amplify across the hotspot and containing 8-base-pair restriction sites (green). *Step 2*: Amplified DNA was digested with the restriction enzyme. *Step 3*: Hotspot molecules were circularized by diluted ligation, bringing flanking SNPs into close proximity. *Step 4*: A second round of PCR was performed using barcoded primers to identify sperm samples from individual mice. *Step 5*: Individual samples were pooled prior to library preparation for high-throughput paired-end sequencing. Each sequence reported the individual mouse that the read belonged to using the unique sequence bar codes, as well as the identity of the SNP to determine if the DNA molecule was of the parental or recombinant genotype.
(EPS)

S2 Fig QTL scans for activity of *Pbx1*. (A) Manhattan plot showing the result of a single QTL genome scan from the N2 backcross for recombination rate at Pbx1. (B) Similar to A showing results of QTL scan for the F2 intercross. (C) A genome scan of *Prdm9* ^*Dom2/Dom2*^ mice did not detect other significant loci contributing to the recombination rate at Pbx1. Manhattan plot of mouse chromosomes showing LOD scores for Pbx1 recombination rate (n = 74 mice).
(EPS)

S3 Fig Relationship between genome-wide H3K4me3 ChIP-seq in HEK293 cells and DMC1 SSDS from men. (A) Venn diagram showing relationships between H3K4me3 ChIP-seq from HEK293 cells either transfected with empty vector, *PRDM9* ^*A*^, or *PRDM9* ^*C*^. Most H3K4me3 peaks found in the empty vector control are shared between all datasets indicating common regulatory locations such as promoters and enhancers. H3K4me3 peaks that are unique to each *PRDM9* allele are considered putative PRDM9 binding sites. Often peak locations do not relate between datasets in a 1-to-1 fashion. For example, 20,696 peaks from the empty vector control overlap with 25,285 PRDM9^A^ and 27,059 PRDM9^C^ H3K4me3 peaks. (B) Method for identifying PRDM9^C^-defined DSB hotspots using DMC1 SSDS ChIP-seq data generated from two individual men (data from [28]). Peaks that are unique to the A/C individual are considered putative PRDM9^C^-defined hotspots. (C) and (D) H3K4me3 ChIP-seq from HEK293 cells identifies approximately one-third of in vivo observed DSB hotspots for both *PRDM9* ^*A*^ (C) and *PRDM9* ^*C*^ (D) alleles.
(EPS)

S4 Fig H3K4me3 at promoters from HEK293 cells lines under different conditions. Promoters show H3K4me3 signal in the empty vector control, highlighting that the absence of H3K4me3 signal at hotspots is due to lack of *Prdm9* expression, not lack of H3K4me3 ChIP (TSS—transcription start sites). (A) qPCR of a control locus at the promoter of GAPDH following H3K4me3 ChIP. The empty vector control shows enrichment of the PRDM9-independent H3K4me3 modification at promoters, while the no-antibody IgG control does not. (B) Heat map showing H3K4me3 signal at promoters aligned to TSS in ChIP data from HEK293 cells. Promoter regions in empty vector control have higher read counts due to fewer genome-wide total peaks, high fraction of reads in peaks (S1 Table), and similar sequencing depth.
(EPS)

S5 Fig Human hotspots used in Fig 3, not previously described, identified using H3K4me3 and DSB. (A) Coverage profile for H3K4me3 and DSB hotspot at Chr 3 114.116 Mb. The Chr 3 114 hotspot shows specific H3K4me3 only after expression of PRDM9^C^ and only has DSBs in the man which carries the C-allele. (B) Similar to A. The hotspot at Chr 3 4.486 Mb is responsive to PRDM9^C^. (C) Coverage profile for H3K4me3 and DSB for hotspot at Chr 3 54.819 Mb. DSBs are found at this hotspot in men either homozygous or heterozygous for the *PRDM9* ^*A*^ allele and shows allele-specific H3K4me3 in HEK293 cells only when *PRDM9* ^*A*^ is expressed. For all hotspots, the PRDM9 motif positions (red bar) and corresponding DNA sequences are indicated, and the locations of DNA primers used for qPCR in Fig 3 are shown in green.
(EPS)

S6 Fig B6-*Prdm9* ^*Dom2/-*^ male mice display meiotic DSB levels comparable to B6. (A) Representative zygonema chromatin spreads prepared from *Prdm9* ^*Dom2/Dom2*^ (B6, upper image) and B6-*Prdm9* ^*Dom2/tm1Ymat*^ (lower image) adult testes, immunolabeled with anti-RAD51/DMC1 (green) to mark programmed DSBs, anti-SYCP3 (red) for staging; DNA is visualized by DAPI (blue). (B) The number of RAD51/DMC1 foci were quantified for *Prdm9* ^*Dom2/Dom2*^ (2 animals, 16 cells) and B6-*Prdm9* ^*Dom2/tm1Ymat*^ (*Dom2/null*, 2 animals, 28 cells). Horizontal bars indicate mean, vertical bars indicate standard deviation.
(EPS)

S1 Table Summary of H3K4me3 ChIP-seq datasets from HEK293 cells and DMC1 SSDS. DMC1 (representing DSBs from human males) data from [28].
(DOCX)

S2 Table Recombination rate at *Ush2a* in pooled sperm samples. *C-B and C-C indicate the recombinant and parental 5’-3’ orientation, respectively, of amplified DNA molecules detected by allele-specific PCR.
(DOCX)

S3 Table Oligonucleotide primers used in this study. (DOCX)

## References

[1] Baudat F, Imai Y, de Massy B (2013) Meiotic recombination in mammals: localization and regulation. Nat Rev Genet 14: 794–806. 10.1038/nrg3573 24136506

[2] Paigen K, Petkov P (2010) Mammalian recombination hot spots: properties, control and evolution. Nat Rev Genet 11: 221–233. 10.1038/nrg2712 20168297 PMC4389181

[3] Axelsson E, Webster MT, Ratnakumar A, Ponting CP, Lindblad-Toh K (2012) Death of PRDM9 coincides with stabilization of the recombination landscape in the dog genome. Genome Res 22: 51–63. 10.1101/gr.124123.111 22006216 PMC3246206

[4] Munoz-Fuentes V, Di Rienzo A, Vila C (2011) Prdm9, a major determinant of meiotic recombination hotspots, is not functional in dogs and their wild relatives, wolves and coyotes. PLoS One 6: e25498 10.1371/journal.pone.0025498 22102853 PMC3213085

[5] Baudat F, Buard J, Grey C, Fledel-Alon A, Ober C, et al (2010) PRDM9 is a major determinant of meiotic recombination hotspots in humans and mice. Science 327: 836–840. 10.1126/science.1183439 20044539 PMC4295902

[6] Myers S, Bowden R, Tumian A, Bontrop RE, Freeman C, et al (2010) Drive against hotspot motifs in primates implicates the PRDM9 gene in meiotic recombination. Science 327: 876–879. 10.1126/science.1182363 20044541 PMC3828505

[7] Parvanov ED, Petkov PM, Paigen K (2010) Prdm9 controls activation of mammalian recombination hotspots. Science 327: 835 10.1126/science.1181495 20044538 PMC2821451

[8] Buard J, Barthes P, Grey C, de Massy B (2009) Distinct histone modifications define initiation and repair of meiotic recombination in the mouse. EMBO J 28: 2616–2624. 10.1038/emboj.2009.207 19644444 PMC2738703

[9] Baker CL, Kajita S, Walker M, Petkov PM, Paigen K (2014) PRDM9 binding organizes hotspot nucleosomes and limits Holliday junction migration. Genome Res 24: 724–732. 10.1101/gr.170167.113 24604780 PMC4009602

[10] Brick K, Smagulova F, Khil P, Camerini-Otero RD, Petukhova GV (2012) Genetic recombination is directed away from functional genomic elements in mice. Nature 485: 642–645. 10.1038/nature11089 22660327 PMC3367396

[11] Smagulova F, Gregoretti IV, Brick K, Khil P, Camerini-Otero RD, et al (2011) Genome-wide analysis reveals novel molecular features of mouse recombination hotspots. Nature 472: 375–378. 10.1038/nature09869 21460839 PMC3117304

[12] Keeney S (2008) Spo11 and the Formation of DNA Double-Strand Breaks in Meiosis. Genome dynamics and stability 2: 81–123. 21927624 10.1007/7050_2007_026 PMC3172816

[13] Hayashi K, Yoshida K, Matsui Y (2005) A histone H3 methyltransferase controls epigenetic events required for meiotic prophase. Nature 438: 374–378. 16292313 10.1038/nature04112

[14] Irie S, Tsujimura A, Miyagawa Y, Ueda T, Matsuoka Y, et al (2009) Single-nucleotide polymorphisms of the PRDM9 (MEISETZ) gene in patients with nonobstructive azoospermia. J Androl 30: 426–431. 10.2164/jandrol.108.006262 19168450

[15] Miyamoto T, Koh E, Sakugawa N, Sato H, Hayashi H, et al (2008) Two single nucleotide polymorphisms in PRDM9 (MEISETZ) gene may be a genetic risk factor for Japanese patients with azoospermia by meiotic arrest. J Assist Reprod Genet 25: 553–557. 10.1007/s10815-008-9270-x 18941885 PMC2593767

[16] Mihola O, Trachtulec Z, Vlcek C, Schimenti JC, Forejt J (2009) A mouse speciation gene encodes a meiotic histone H3 methyltransferase. Science 323: 373–375. 10.1126/science.1163601 19074312

[17] Berg IL, Neumann R, Lam KW, Sarbajna S, Odenthal-Hesse L, et al (2010) PRDM9 variation strongly influences recombination hot-spot activity and meiotic instability in humans. Nat Genet 42: 859–863. 10.1038/ng.658 20818382 PMC3092422

[18] Berg IL, Neumann R, Sarbajna S, Odenthal-Hesse L, Butler NJ, et al (2011) Variants of the protein PRDM9 differentially regulate a set of human meiotic recombination hotspots highly active in African populations. Proc Natl Acad Sci U S A 108: 12378–12383. 10.1073/pnas.1109531108 21750151 PMC3145720

[19] Hinch AG, Tandon A, Patterson N, Song Y, Rohland N, et al (2011) The landscape of recombination in African Americans. Nature 476: 170–175. 10.1038/nature10336 21775986 PMC3154982

[20] Grey C, Barthes P, Chauveau-Le Friec G, Langa F, Baudat F, et al (2011) Mouse PRDM9 DNA-binding specificity determines sites of histone H3 lysine 4 trimethylation for initiation of meiotic recombination. PLoS Biol 9: e1001176 10.1371/journal.pbio.1001176 22028627 PMC3196474

[21] Groeneveld LF, Atencia R, Garriga RM, Vigilant L (2012) High diversity at PRDM9 in chimpanzees and bonobos. PLoS One 7: e39064 10.1371/journal.pone.0039064 22768294 PMC3388066

[22] Auton A, Fledel-Alon A, Pfeifer S, Venn O, Segurel L, et al (2012) A fine-scale chimpanzee genetic map from population sequencing. Science 336: 193–198. 10.1126/science.1216872 22422862 PMC3532813

[23] Schwartz JJ, Roach DJ, Thomas JH, Shendure J (2014) Primate evolution of the recombination regulator PRDM9. Nat Commun 5: 4370 10.1038/ncomms5370 25001002 PMC4110516

[24] Sandor C, Li W, Coppieters W, Druet T, Charlier C, et al (2012) Genetic variants in REC8, RNF212, and PRDM9 influence male recombination in cattle. PLoS Genet 8: e1002854 10.1371/journal.pgen.1002854 22844258 PMC3406008

[25] Steiner CC, Ryder OA (2013) Characterization of Prdm9 in equids and sterility in mules. PLoS One 8: e61746 10.1371/journal.pone.0061746 23613924 PMC3632555

[26] Parvanov ED, Ng SH, Petkov PM, Paigen K (2009) Trans-regulation of mouse meiotic recombination hotspots by Rcr1. PLoS Biol 7: e36 10.1371/journal.pbio.1000036 19226189 PMC2642880

[27] Neumann R, Jeffreys AJ (2006) Polymorphism in the activity of human crossover hotspots independent of local DNA sequence variation. Hum Mol Genet 15: 1401–1411. 16543360 10.1093/hmg/ddl063

[28] Pratto F, Brick K, Khil P, Smagulova F, Petukhova GV, et al (2014) DNA recombination. Recombination initiation maps of individual human genomes. Science 346: 1256442 10.1126/science.1256442 25395542 PMC5588152

[29] Segurel L, Leffler EM, Przeworski M (2011) The case of the fickle fingers: how the PRDM9 zinc finger protein specifies meiotic recombination hotspots in humans. PLoS Biol 9: e1001211 10.1371/journal.pbio.1001211 22162947 PMC3232208

[30] Baker CL, Kajita S, Walker M, Saxl RL, Raghupathy N, et al (2015) PRDM9 Drives Evolutionary Erosion of Hotspots in Mus musculus through Haplotype-Specific Initiation of Meiotic Recombination. PLoS Genet 11: e1004916 10.1371/journal.pgen.1004916 25568937 PMC4287450

[31] Billings T, Parvanov ED, Baker CL, Walker M, Paigen K, et al (2013) DNA binding specificities of the long zinc-finger recombination protein PRDM9. Genome Biol 14: R35 10.1186/gb-2013-14-4-r35 23618393 PMC4053984

[32] Hinch AG, Altemose N, Noor N, Donnelly P, Myers SR (2014) Recombination in the human Pseudoautosomal region PAR1. PLoS Genet 10: e1004503 10.1371/journal.pgen.1004503 25033397 PMC4102438

[33] Eram MS, Bustos SP, Lima-Fernandes E, Siarheyeva A, Senisterra G, et al (2014) Trimethylation of histone H3 lysine 36 by human methyltransferase PRDM9 protein. J Biol Chem 289: 12177–12188. 10.1074/jbc.M113.523183 24634223 PMC4002121

[34] Sun F, Fujiwara Y, Reinholdt LG, Hu J, Saxl RL, et al (2015) Nuclear localization of PRDM9 and its role in meiotic chromatin modifications and homologous synapsis. Chromosoma. 10.1007/s00412-015-0511-3 PMC4550572 25894966

[35] Fairfield H, Gilbert GJ, Barter M, Corrigan RR, Curtain M, et al (2011) Mutation discovery in mice by whole exome sequencing. Genome Biol 12: R86 10.1186/gb-2011-12-9-r86 21917142 PMC3308049

[36] Flachs P, Mihola O, Simecek P, Gregorova S, Schimenti JC, et al (2012) Interallelic and intergenic incompatibilities of the Prdm9 (Hst1) gene in mouse hybrid sterility. PLoS Genet 8: e1003044 10.1371/journal.pgen.1003044 23133405 PMC3486856

[37] Weiss J, Hurley LA, Harris RM, Finlayson C, Tong M, et al (2012) ENU mutagenesis in mice identifies candidate genes for hypogonadism. Mamm Genome 23: 346–355. 10.1007/s00335-011-9388-5 22258617 PMC3358541

[38] Flachs P, Bhattacharyya T, Mihola O, Pialek J, Forejt J, et al (2014) Prdm9 incompatibility controls oligospermia and delayed fertility but no selfish transmission in mouse intersubspecific hybrids. PLoS One 9: e95806 10.1371/journal.pone.0095806 24756080 PMC3995920

[39] Dzur-Gejdosova M, Simecek P, Gregorova S, Bhattacharyya T, Forejt J (2012) Dissecting the genetic architecture of F1 hybrid sterility in house mice. Evolution 66: 3321–3335. 10.1111/j.1558-5646.2012.01684.x 23106700

[40] Bhattacharyya T, Gregorova S, Mihola O, Anger M, Sebestova J, et al (2013) Mechanistic basis of infertility of mouse intersubspecific hybrids. Proc Natl Acad Sci U S A 110: E468–477. 10.1073/pnas.1219126110 23329330 PMC3568299

[41] Fog CK, Galli GG, Lund AH (2012) PRDM proteins: important players in differentiation and disease. Bioessays 34: 50–60. 10.1002/bies.201100107 22028065

[42] Hohenauer T, Moore AW (2012) The Prdm family: expanding roles in stem cells and development. Development 139: 2267–2282. 10.1242/dev.070110 22669819

[43] Huang S, Shao G, Liu L (1998) The PR domain of the Rb-binding zinc finger protein RIZ1 is a protein binding interface and is related to the SET domain functioning in chromatin-mediated gene expression. J Biol Chem 273: 15933–15939. 9632640 10.1074/jbc.273.26.15933

[44] Davis CA, Haberland M, Arnold MA, Sutherland LB, McDonald OG, et al (2006) PRISM/PRDM6, a transcriptional repressor that promotes the proliferative gene program in smooth muscle cells. Molecular and cellular biology 26: 2626–2636. 16537907 10.1128/MCB.26.7.2626-2636.2006 PMC1430312

[45] Friedman JR, Fredericks WJ, Jensen DE, Speicher DW, Huang XP, et al (1996) KAP-1, a novel corepressor for the highly conserved KRAB repression domain. Genes & development 10: 2067–2078. 8769649 10.1101/gad.10.16.2067

[46] Urrutia R (2003) KRAB-containing zinc-finger repressor proteins. Genome Biol 4: 231 14519192 10.1186/gb-2003-4-10-231 PMC328446

[47] Edelstein LC, Collins T (2005) The SCAN domain family of zinc finger transcription factors. Gene 359: 1–17. 16139965 10.1016/j.gene.2005.06.022

[48] Buard J, Rivals E, Dunoyer de Segonzac D, Garres C, Caminade P, et al (2014) Diversity of Prdm9 Zinc Finger Array in Wild Mice Unravels New Facets of the Evolutionary Turnover of this Coding Minisatellite. PLoS One 9: e85021 10.1371/journal.pone.0085021 24454780 PMC3890296

[49] Kono H, Tamura M, Osada N, Suzuki H, Abe K, et al (2014) Prdm9 polymorphism unveils mouse evolutionary tracks. DNA Res 21: 315–326. 10.1093/dnares/dst059 24449848 PMC4060951

[50] Shultz KL, Donahue LR, Bouxsein ML, Baylink DJ, Rosen CJ, et al (2003) Congenic strains of mice for verification and genetic decomposition of quantitative trait loci for femoral bone mineral density. J Bone Miner Res 18: 175–185. 12568393 10.1359/jbmr.2003.18.2.175

[51] Anderson LK, Reeves A, Webb LM, Ashley T (1999) Distribution of crossing over on mouse synaptonemal complexes using immunofluorescent localization of MLH1 protein. Genetics 151: 1569–1579. 10101178 10.1093/genetics/151.4.1569 PMC1460565

[52] Broman KW, Wu H, Sen S, Churchill GA (2003) R/qtl: QTL mapping in experimental crosses. Bioinformatics 19: 889–890. 12724300 10.1093/bioinformatics/btg112

[53] Colot HV, Park G, Turner GE, Ringelberg C, Crew CM, et al (2006) A high-throughput gene knockout procedure for Neurospora reveals functions for multiple transcription factors. Proc Natl Acad Sci U S A 103: 10352–10357. 16801547 10.1073/pnas.0601456103 PMC1482798

[54] Li H, Durbin R (2009) Fast and accurate short read alignment with Burrows-Wheeler transform. Bioinformatics 25: 1754–1760. 10.1093/bioinformatics/btp324 19451168 PMC2705234

[55] Zhang Y, Liu T, Meyer CA, Eeckhoute J, Johnson DS, et al (2008) Model-based analysis of ChIP-Seq (MACS). Genome Biol 9: R137 10.1186/gb-2008-9-9-r137 18798982 PMC2592715

[56] Bailey TL, Boden M, Buske FA, Frith M, Grant CE, et al (2009) MEME SUITE: tools for motif discovery and searching. Nucleic Acids Res 37: W202–208. 10.1093/nar/gkp335 19458158 PMC2703892

[57] Ross-Innes CS, Stark R, Teschendorff AE, Holmes KA, Ali HR, et al (2012) Differential oestrogen receptor binding is associated with clinical outcome in breast cancer. Nature 481: 389–393. 10.1038/nature10730 22217937 PMC3272464

[58] Ye T, Krebs AR, Choukrallah MA, Keime C, Plewniak F, et al (2011) seqMINER: an integrated ChIP-seq data interpretation platform. Nucleic Acids Res 39: e35 10.1093/nar/gkq1287 21177645 PMC3064796

[59] Quinlan AR, Hall IM (2010) BEDTools: a flexible suite of utilities for comparing genomic features. Bioinformatics 26: 841–842. 10.1093/bioinformatics/btq033 20110278 PMC2832824

