# Supplementary material for: Multimer Formation Explains Allelic Suppression of PRDM9 Recombination Hotspots
Source: PLoS Genet. 2015 Sep 14;11(9):e1005512. doi: 10.1371/journal.pgen.1005512 (PMC4569383; doi:10.1371/journal.pgen.1005512)
Supplement: S1 Table — DMC1 (representing DSBs from human males) data from [28]. (DOCX) [file pgen.1005512.s007.docx]

**Supplemental Table S1.** Summary of H3K4me3 ChIP-seq from HEK293 cells and analysis of DMC1 SSDS (representing DSBs from human samples). DMC1 SSDS reads from [28].

|  | Sample | Tissue/  Cells | Antibody | Allele | # of peaks  (e-5) | ChIP reads | Control reads | FRiP* |
| --- | --- | --- | --- | --- | --- | --- | --- | --- |
| 1 | pCB53 | HEK293 | H3K4me3 | FLAG-PRDM9^A^ | 72,681 | 28,049,772 | 44,354,283 | 29.2% |
| 2 | pCB51 | HEK293 | H3K4me3 | FLAG-PRDM9^C^ | 70,171 | 30,597,602 | 29,202,880 | 22.7% |
| 3 | pCEP4 | HEK293 | H3K4me3 | Empty Vector | 33,988 | 40,406,323 | 41,659,579 | 73.2% |
| 4 | pCB56 | HEK293 | H3K4me3 | FLAG-PRDM9^C-G278A^ | 39,392 | 40,086,098 | 47,075,514 | 82.1% |
| 5 | pCB56 + pCB48 | HEK293 | H3K4me3 | FLAG-PRDM9^C-G278A^ +  V5-PRDM9^A^ | 83,236 | 35,759,047 | 41,963,008 | 40.5% |
| 6 | A/A_1_ | Testis | DMC1 | PRDM9^A^ | 34,303 |  |  |  |
| 7 | A/C | Testis | DMC1 | PRDM9^A/C^ | 49,563 |  |  |  |

* Fraction of Reads in Peaks
