# Supplementary material for: Multimer Formation Explains Allelic Suppression of PRDM9 Recombination Hotspots
Source: PLoS Genet. 2015 Sep 14;11(9):e1005512. doi: 10.1371/journal.pgen.1005512 (PMC4569383; doi:10.1371/journal.pgen.1005512)
Supplement: S2 Table — *C-B and C-C indicate the recombinant and parental 5’-3’ orientation, respectively, of amplified DNA molecules detected by allele-specific PCR. (DOCX) [file pgen.1005512.s008.docx]

| Cross | *Prdm9*  alleles | C-B* mol/µg | C-C* mol/µg | Sperm Recombination Rate  (cM) | Progeny Recombination Rate  (cM) |
| --- | --- | --- | --- | --- | --- |
| B6 x  B6.CAST-1T | *Dom2/*  *Dom2* | 54 | 6,588 | 0.82 | 0.93 |
| B6-*Prdm9^Dom2/-^* x  B6.CAST-1T | *Dom2/-* | 63 | 7,332 | 0.85 | - |
| B6 x CAST | *Dom2/Cst* | 0 | 5,893 | 0.00 | 0.00 |

**Supplemental Table S2**. Recombination rate at *Ush2a* in pooled sperm DNA samples from F1 hybrid male mice.

*C-B and C-C indicate recombinant and parental orientation, respectively, of amplified DNA molecules detected by hotspot allele-specific PCR.
