# Supplementary material for: Multimer Formation Explains Allelic Suppression of PRDM9 Recombination Hotspots
Source: PLoS Genet. 2015 Sep 14;11(9):e1005512. doi: 10.1371/journal.pgen.1005512 (PMC4569383; doi:10.1371/journal.pgen.1005512)
Supplement: S3 Table — (DOCX) [file pgen.1005512.s009.docx]

**Supplemental Table S3.** Primers and synthetic oligonucleotides used in this study

| Name | Sequence |
| --- | --- |
| Primers for measuring recombination rate at Pbx1 | |
| Pbx1_1F | ATTAGCGGCCGCCAGACTCTAAACCTGTCAAAGGC |
| Pbx1_1R | ATTAGCGGCCGCGAATGATGACAGCCAGCTGAGG |
| Pbx1_2F | *NNNNNXXXXXXXXGAACCTACTCTGATATTGTCTTTCTC |
| Pbx1_2R | *NNNNNXXXXXXXXGTCCTACCTTTGTAAGTTCTCCAT |
| * NNNNN represent random nucleotides to assist sequence complexity for Illumina high-throughput sequence; XXXXXXXX represent sequences of various 8-mer used for DNA barcoding different samples | |
| Primers for measuring recombination at Ush2a | |
| Ush2a-f1CAST | GCCTGAAATAAAGAGGTTACAGT |
| Ush2a-r1CAST | ATCACCATCCAACTCCACTT |
| Ush2a-r1B6 | ATCACCATCCAACTCCACTC |
| Ush2a-f2CAST | GTTGTGAATCAGGTGATTGGC |
| Ush2a-r2CAST | GGAGATAATCTCATTTATCTTAATG |
| Ush2a-r2B6 | GGAGATAATCTCATTTATCTTAATA |
| Primers for cloning *hsPRDM9* | |
| hPrdm9_V5_F | ATTAGGTACCGCTAGCCACCATGGGTAAGCCTATCCCTAACCCTCTCCTCGGTCTCGATTCTACGAGCCCTGAAAAGTCCCAAG |
| hPrdm9_V5_R | ATTAGGATCCAAGCTTTTACTCATCCTCCCTGCAG |
| hFLAG_F | ACTCACTATAGGGCGAATTGGGTACCAGCTGCTAGCCACCATGCATCACCATCATCACCACGGUGGCGGUGACTACAAAGACCATGATGG |
| hFLAG_R | TGGGCTCTCCTCTTGGGACTTTTCAGGGCTTTTATCGTCGTCATCTTTGT |
| hsG278A | CGTCTTCTGTAATTCGGGCCTCATAAGGGCCAAAG |
| hsG278A_anti | CTTTGGCCCTTATGAGGCCCGAATTACAGAAGACG |
| HPRDM9_E12 | GAAAGAGCTCATGGCAGGGAGAGAACCAAAGCCAGAGATCCATCC |
| HPRDM9r | ATGCAAGCTTTTACTCATCCTCCCTGCAGAC |
| Primers for quantitative PCR | |
| H3K4me3 ChIP in HEK293 cells | |
| S_2kb_LF | TAATGTGGGGATGGGGTTTA |
| S_2kb_LR | TACAAGCCATGGCAGGATTT |
| S_750bp_LF | CATGAGAAGAGGAAATGGCTTT |
| S_750bp_LR | TTTGACAGATTACCCCAGACC |
| S_300bpL_F | CCACTTGCAATGAGGCAATA |
| S_300bpL_R | TCATGTTATCTGGGAATAGAAACAG |
| S_BS1_F | AAGAAGTGCAAACTTATGCTCTCA |
| S_BS1_R | CACGAACATGAAATGTTTTTCC |
| S_BS2_RF | TGGTCAAGTGATTTTCACCAAG |
| S_BS2_RR | ATGTAGTTGTCCCAGCACCA |
| S_1kb_RF | AAGCAATTTGGCATTCATCG |
| S_1kb_RR | CACATGGAAGTTTTTGTGTGG |
| S_2kb_RF | TTTCTACTTGATCCTGCATCTG |
| S_2kb_RR | TCCCTTCAACAAAATCGTGTC |
| F_1kbL_F | AAAGGGAAGAGGGGCAAGTA |
| F_1kbL_R | GCGCGTGCCTGTATTTTTAT |
| F_500bp_F | TGATGAGAGTCCAGGCTGTG |
| Primers for quantitative PCR cont. | |
| F_500bp_R | CCTGTGCCCCAGATACTCAT |
| F_Center_F | TGTCTTTGGCTTACCCGAAC |
| F_Center_R | TGTGGAGCATGAGATGAAGG |
| F_1kbR_F | TGAGCACCTGACTGTGAAGG |
| F_1kbR_R | ACAACTGCCACCAGCAAGTT |
| 5A_3kbL_F | CCTGCTCCCTTGTGTTTAGG |
| 5A_3kbL_R | TCTGTGGGGAGTCCTATGATG |
| 5A_1kbL_F | AACGCCTCCTTGTATTTAGACG |
| 5A_1kbL_R | GACATGCCTAGCGTTCCATT |
| 5A_300bpL_F | CTGTGCTGTGGTTCATCCAT |
| 5A_300bpL_R | CTGGGTCTGGTGTAGGAGGA |
| 5A_Center_F | ATTGCTAGAAAGGCGTGTGC |
| 5A_Center_R | TTGCTAGGCATGTGAAATGG |
| 5A_700bpR_F | CTCAGAAACCTTTGCCCAAC |
| 5A_700bpR_R | CAGAAGACGGGAAAAAGCTG |
| 5A_1kbR_F | GTGGGCACTGATGCTCTGTA |
| 5A_1kbR_R | CTCCCTCCAGTAACCCCACT |
| 5A_3kbR_F | TTTAAACGGTGGAAGCGTCT |
| 5A_3kbR_R | GCGACAGTCACACGTTTAGC |
| 22A_3kbL_F | TTCATGGGGAAGTCAGGAAG |
| 22A_3kbL_R | TGACAGAAGCCTCTCCTGGT |
| 22A_1kbL_F | TCAGATGGAAAGGTGGCATT |
| 22A_1kbL_R | GTCCTCTTTCTTCCCCACCT |
| 22A_400bpL_F | GATGGGAAGGGGAAAATGAT |
| 22A_400bpL_R | AACAGGCTTCCACCCTACCT |
| 22A_Center_F | GAAGCAAGCTGGGTTCAGTC |
| 22A_Center_R | TTGCCAAGTGTAGCTGTCAGT |
| 22A_300bpR_F | AGATGCCCTGATTTCATTCC |
| 22A_300bpR_R | TTGTGGACTGACTGGGTGTG |
| 22A_1kbR_F | AGTGAGAATGGGTGCCAGAC |
| 22A_1kbR_R | CATTTTGCAAGAGAGTGCTCA |
| 22A_3kbR_F | ACCCAATCTGACTGGTGTCC |
| 22A_3kbR_R | CTTCACTTGGCTGACCCTCT |
| FLAG ChIP for PRDM9 in HEK293 cells | |
| 5A_Center_F | ATTGCTAGAAAGGCGTGTGC |
| 5A_Center_R | TTGCTAGGCATGTGAAATGG |
| C_chr3_114_1F | TGTTCCACACCCAGCTATTG |
| C_chr3_114_1R | ATCATGGGGCAAGATCAAAC |
| C_chr3_44_F | CACTGGGGATGGTAGCATTAG |
| C_chr3_44_R | TCAAGGACACAGGGGATGAT |
| A_chr3_54_1F | AACTGCACAGCTGCAAACAC |
| A_chr3_54_1R | TATCCCAACCAATCCCATGT |
